# Inhibiting Bone Morphogenetic Protein 4 Type I Receptor Signaling Promotes Remyelination by Potentiating Oligodendrocyte Differentiation

**DOI:** 10.1523/ENEURO.0399-18.2019

**Published:** 2019-05-14

**Authors:** Alistair E. Govier-Cole, Rhiannon J. Wood, Jessica L. Fletcher, David G. Gonsalvez, Daniel Merlo, Holly S. Cate, Simon S. Murray, Junhua Xiao

**Affiliations:** Department of Anatomy and Neuroscience, University of Melbourne, Parkville 3010, Victoria, Australia

**Keywords:** BMP4, BMPRIA, demyelination, oligodendrocyte

## Abstract

Blocking inhibitory factors within CNS demyelinating lesions is regarded as a promising strategy to promote remyelination. Bone morphogenetic protein 4 (BMP4) is an inhibitory factor present in demyelinating lesions. Noggin, an endogenous antagonist to BMP, has previously been shown to increase the number of oligodendrocytes and promote remyelination *in vivo.* However, it remains unclear how BMP4 signaling inhibits remyelination. Here we investigated the downstream signaling pathway that mediates the inhibitory effect that BMP4 exerts upon remyelination through pharmacological and transgenic approaches. Using the cuprizone mouse model of central demyelination, we demonstrate that selectively blocking BMP4 signaling via the pharmacological inhibitor LDN-193189 significantly promotes oligodendroglial differentiation and the extent of remyelination *in vivo*. This was accompanied by the downregulation of transcriptional targets that suppress oligodendrocyte differentiation. Further, selective deletion of BMP receptor type IA (BMPRIA) within primary mouse oligodendrocyte progenitor cells (OPCs) significantly enhanced their differentiation and subsequent myelination *in vitro*. Together, the results of this study identify that BMP4 signals via BMPRIA within OPCs to inhibit oligodendroglial differentiation and their capacity to myelinate axons, and suggest that blocking the BMP4/BMPRIA pathway in OPCs is a promising strategy to promote CNS remyelination.

## Visual Abstract

**Figure:**
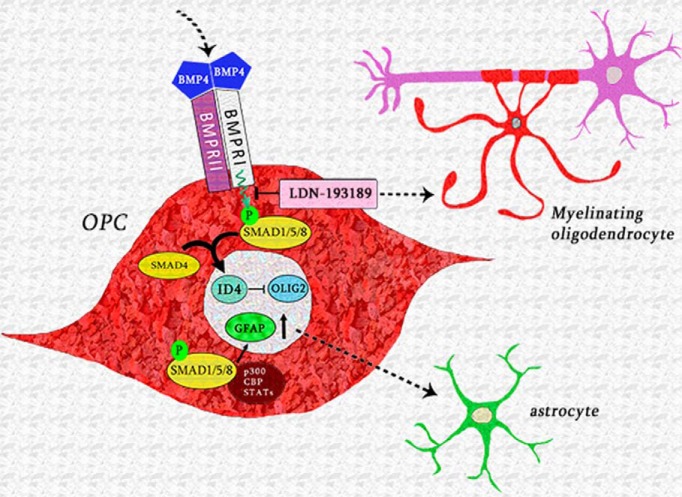

## Significance Statement

Blocking inhibitory factors within central demyelinating lesions is a promising strategy to promote remyelination. Previous studies have established that exogenous bone morphogenetic protein (BMPs) inhibit oligodendrocyte differentiation during CNS development and after injury. Here, we demonstrate that blocking endogenous BMP4 signaling via a selective pharmacological approach promotes oligodendroglial differentiation and the rate of remyelination after a central demyelinating insult *in vivo.* Using *in vitro* analysis, we identify that oligodendrocyte progenitor cell (OPC)-expressed BMP Type I receptors mediate this effect. Together, our data propose that blocking the BMP4 signaling pathway at the Type I receptors in OPCs is a promising strategy to promote CNS remyelination.

## Introduction

In central demyelinating diseases such as multiple sclerosis (MS), oligodendrocytes (OLs) are targeted through inflammatory activity and the myelin sheath surrounding axons is degraded (Noseworthy, 1999; Weiner, 2009). The degree of remyelination within demyelinating lesions is variable; although MS lesions remyelinate relatively efficiently early on in disease, at later stages many lesions remain chronically demyelinated (Trapp and Nave, 2008). These chronically demyelinated lesions typically contain oligodendrocyte progenitor cells (OPCs) and premyelinating OLs that have “stalled” in their differentiation, implicating blocked OL differentiation as a major contributing factor to remyelination failure (Chang et al., 2002; Kuhlmann et al., 2008). Although the full complement of factors that inhibit OL differentiation and remyelination in the context of MS are yet to be completely elucidated, they most likely include a variety of inhibitory signals present within the lesion environment as well as an absence of positive signals (Fancy et al., 2011; Kotter et al., 2011; Franklin et al., 2012). Thus, blocking the action of inhibitory factors is regarded as a leading strategy to promote endogenous CNS remyelination (Franklin and Gallo, 2014).

The bone morphogenetic proteins (BMPs) are a group of secreted proteins that are part of the larger transforming growth factor-β (TGF-β) superfamily (Chen et al., 2004) and play critical roles in neural development and gliogenesis (Bond et al., 2012; Cole et al., 2016). Of the 20 BMPs, BMP4 has a prominent role in promoting astroglial and inhibiting oligodendroglial specification (Gomes et al., 2003; Grinspan, 2015). *In vitro*, BMP4 exerts stage-specific inhibitory effects on OPCs (Grinspan et al., 2000), in particular inhibiting the production of myelin proteins by immature OLs (See et al., 2004). *In vivo,* transgenic overexpression of BMP4 led to an increase in the number of astrocytes and a decrease in the number of oligodendrocytes in the murine CNS (Gomes et al., 2003). In the context of demyelinating disease, BMP4 mRNA is detected in human demyelinated MS lesions (Deininger et al., 1995), and is expressed by astrocytes, microglia, and infiltrating immune cells (Harnisch et al., 2019). Astrocytes also express a high level of BMP4 in chronic lesions that have failed to remyelinate (Harnisch et al., 2019). Through using the cuprizone-induced murine model of CNS demyelination, we have previously found that BMP4 mRNA is upregulated in the mouse corpus callosum (CC) following a demyelinating insult *in vivo* (Cate et al., 2010). Furthermore, we demonstrated that inhibiting BMP4 signaling following cuprizone-induced CNS demyelination via infusion of its extracellular antagonist noggin resulted in more mature oligodendrocytes and more remyelinated axons (Cate et al., 2010; Sabo et al., 2011). However, in addition to BMP4, noggin also inhibits other BMPs such as BMP2, 7, 13, and 14 (Krause et al., 2011). Due to the promiscuous inhibitory effect of noggin and the potential effects it exerted on oligodendroglia, astrocytes, and microglia, the precise influence that the inhibition of BMP4 exerts on remyelination and the cell type mediating the effect remain unclear.

BMP4 signals through membrane-bound receptor complexes composed of two type I receptors and two type II receptors. While several type I receptors exist, BMP4 has the greatest affinity for the BMP type I receptors BMPRIA (also known as ALK3) and BMPRIB (also known as ALK6; Liu et al., 1995; Knaus and Sebald, 2001). In the presence of BMP4, BMPRIA and BMPRIB initiate signaling via phosphorylation of SMAD1, SMAD5, and SMAD8 (Cuny et al., 2008) and the pharmacological inhibitor LDN-193189 selectively blocks phosphorylation SMAD1/5/8 (Cuny et al., 2008). To specifically interrogate the influence that BMP4 signaling exerted on remyelination, we infused LDN-193189 into the brain following cuprizone-induced demyelination and found it significantly enhanced oligodendroglial differentiation and their subsequent remyelination following the demyelinating insult *in vivo*. This finding is also supported *in vitro* in which LDN-193189 significantly enhanced OPC differentiation and myelination. Further, by using a tamoxifen-dependent inducible conditional knockout (KO) mouse strategy (*Pdgfra-CreER^T2^*::*Bmpr1a* ^fl/fl^) to specifically ablate BMPRIA expression within OPCs, we identified that selectively deleting of BMPRIA in OPCs significantly potentiated their differentiation into mature oligodendrocytes and increased myelin formation *in vitro*. Together, our findings indicate that BMP4 acts on OPC-expressed BMPRIA receptors to inhibit oligodendroglial differentiation and myelination, and that blocking BMPRIA signaling OPCs is a promising strategy to promote CNS remyelination.

## Materials and Methods

### Animals and reagents

All animal procedures were performed in accordance with the Florey Institute of Neuroscience and Mental Health Animal Ethics Committee regulations. Female mice (7–8 weeks old) were used for *in vivo* cuprizone experiments and postnatal day 5 (P5) to P7 mice of either sex were used for *in vitro* experiments. C57BL/6 mice were purchased from the Animal Resource Center (Canning Vale, WA, Australia). *Pdgfra-CreER^T2^*::*Bmpr1a* ^fl/fl^ mice were generated by crossing *Pdgfra-CreER^T2^* mouse line (provided by Dr. Kaylene Young, University of Tasmania, Hobart, TAS, Australia; Rivers et al., 2008) with *Bmpr1a* ^fl/fl^ mouse colony (also known as *Alk3^fl/fl^*; provided by Professor Yuji Mishina, University of Michigan, Ann Arbor, MI; Mishina et al., 2002). *Pdgfra-CreER^T2^*::*Bmpr1a* ^fl/fl^ mice have a tamoxifen-inducible deletion of the *Bmpr1a* allele from the start of the sequence to the end of exon 2, rendering it untranscribable (Mishina et al., 1995). All animals used for this study were bred at the Core Animal Services facility of the Florey Institute of Neuroscience and Mental Health. All chemicals were obtained from Sigma-Aldrich, unless otherwise indicated.

### Cuprizone protocol

Cuprizone-mediated demyelination was induced by feeding 8- to 10-week-old female mice (C57BL/6) powdered feed (Barastoc) containing 0.2% cuprizone (w/w: bis-cyclohexanoneoxaldihydrazone) for 5 weeks, as previously described (Cate et al., 2010; Sabo et al., 2011). Mice were then returned to a normal diet for either 0 or 1 week, according to the experimental paradigm. During the 5 week demyelination phase, feed was refreshed every 3 d, with ∼20 g provided per mouse for this period. Mice were weighed daily to monitor extreme fluctuations in weight and to ensure no mouse lost >15% of its initial weight during the protocol. Unchallenged control mice were fed identical feed without added cuprizone.

### Intracerebroventricular infusion

Following cuprizone feeding, animals received either LDN-193189 (400 ng/d; Stemgent) or artificial CSF (aCSF) via intracerebroventricular osmotic pumps (catalog #1007D, Alzet). The concentration of LDN-193189 was based on our previous study using noggin (Sabo et al., 2011). Mice were deeply anaesthetized using 2.5% isoflurane and attached to a stereotactic frame. The scalp was cut sagittal to the cervical spine. The pumps were used in conjunction with Alzet Brain Infusion Kit III to implant a cannula into an entry point drilled 0.5 mm anterior to bregma, 0.7 mm laterally from the longitudinal midline and at a depth of ∼1-2 mm. Canullae were fused to the skull using Araldite, and the incision was sutured with Vicryl veterinary sutures and disinfected using Betadine iodine solution. Mice were allowed to recover for >30 min at 30°C before returning to the cage. Mice were monitored daily to observe any symptoms of distress or infection. After 7 d of continuous infusion, animals were killed and the brain removed for immunohistochemical and histologic analysis.

### Post-cuprizone tissue collection

Following cuprizone withdrawal, mice were transcardially perfused using 0.1 m mouse tonicity PBS (MT-PBS) as a buffer and 4% PFA (in MT-PBS, 15 ml/mouse) as a fixative. Brains were dissected and postfixed overnight with 4% PFA in MT-PBS and rinsed the following day with MT-PBS before being cut coronally into 1 mm sections. For electron microscopy, sections containing the most caudal region of the CC (approximately −2.12 mm from bregma) were trimmed to expose the splenium of the caudal CC and placed in Karnovsky’s buffer (4% PFA, 2.5% glutaraldehyde in 0.1 m sodium cacodylate) overnight before being rinsed three times in 0.1 m sodium cacodylate. For immunohistochemical analyses, sections containing the caudal corpus callosum (approximately −1.12 mm from bregma) were placed in 30% sucrose (in MT-PBS with 0.1% sodium azide) overnight. Sucrose-treated sections were frozen in Tissue Tek Optimum Cutting Temperature (O.C.T., Sakura) solution using chilled isopentane and stored at −80°C.

### Immunohistochemistry

Coronal brain sections were cut at 10 or 12 μm thin and blocked for 1 h in antibody diluent (10% normal goat serum, 0.3% Triton X-100 in MT-PBS) at room temperature (RT) before exposure to primary antibodies diluted in antibody diluent overnight at 4°C. The following primary antibodies were used at a dilution of 1:200: rat anti-myelin basic protein (MBP; catalog #MAB386, Abcam), rabbit anti-OLIG2 (catalog #ab9610, Millipore), rat anti-CC1/APC (catalog #D35078, Calbiochem), mouse anti-platelet-derived growth factor receptor α (PDGFRα; catalog #AF1062, R&D Systems), mouse anti-glial fibrillary acidic protein (GFAP; catalog #MAB360, Millipore), and goat anti-IBA-1 (ionized calcium binding adaptor molecule 1; catalog #ab5076, Abcam). Cryosections were then rinsed with MT-PBS three times for ∼5 min followed by the appropriate fluorophore-conjugated secondary antibodies (all 1:500 in antibody diluent; Thermo Fisher Scientific) for 60 min at RT. Sections were rinsed twice in MT-PBS before adding Hoechst (1:10,000 in MT-PBS; catalog #33342, Invitrogen) for 10 min. Cryosections were rinsed twice in MT-PBS, and a coverslip was mounted with Cytomation fluorescence mounting medium (Dako). Six sections per animal from a minimum of three animals per group were analyzed, and images captured by a Carl Zeiss LSM 780 confocal fluorescent microscopy. All images were acquired using the same settings and were analyzed by an operator blinded to conditions using FIJI (ImageJ 1.51K, National Institutes of Health) software (Schindelin et al., 2012). For OLIG2^+^/CC1^+^/PDGFRα^+^ cell counts, cells were counted from the entire visible corpus callosum per image field with the same size of area. For MBP immunostaining, a central area of 200 μm^2^ was measured for integrated density (the product of the mean gray value of each pixel, ranging from 0 to 255, and the total area) using the “Measure” function in FIJI. For GFAP and IBA-1 immunostaining, the entire corpus callosum was measured using the “Trace” function.

### Spectral confocal reflection microscopy

Spectral confocal reflection (SCoRe) imaging was performed on brain sections to assess the extent of myelin damage in cuprizone mice using published methods (Schain et al., 2014; Gonsalvez et al., 2017a,b). Briefly, mice were perfused with 4% PFA, and their brains were dissected, frozen, and cryosectioned at 12 μm. Coronal sections of caudal brains were imaged via a Zeiss 780 LSM Confocal Microscope with a water-immersion objective [Zeiss W Plan-Apochromat 20×/1.0 numerical aperture (NA) differential interference contrast M27, 70 mm] using 458, 561, and 633 nm laser wavelength through the Tunable Lazer In Tune 488-640 filter/splitter wheel and a 20/80 partially reflective mirror. The reflected light was collected using three photodetectors set to collect light through narrow bands defined by prism and mirror-sliders, centered around the laser wavelengths 488, 561, and 633 nm. Sections were immersed in MT-PBS and a 20× dipping objective was equipped before imaging. The midline corpus callosum was located, and a 3 × 2 tile scan image was taken of each section. The channels from each photodetector were then additively combined as a one-color composite. Myelinated area was calculated using ImageJ by first applying a *Z*-stack transformation and then setting a threshold of 50 pixels. Measurements of the resulting area were obtained with the “Measure” function and divided by the total area of the region of interest (ROI). The percentage area of positive signal was computed for each image. For quantification, a minimum three separate ROIs per image and three images per tile (using a 20×/1.0 NA objective at a *z*-depth 4 μm from the tissue surface) per treatment group were used and statistically analyzed.

### Transmission electron microscopy

Mouse caudal CC samples were embedded in resin for 5 d before trimming and sectioning using an ultramicrotome. Semi-thin sections (0.5 μm) were taken and imaged using toluene blue staining to identify ROI. Ultra-thin sections (70 nm) were then taken and imaged using a transmission electron microscopy (TEM). Images were taken at 5000× and 10,000× magnification per animal using a JEOL 1011 transmission electron microscope. Three 10,000× images were taken per hexagonal bounding grid corresponding to a size of 250 μm^2^, with six distinct fields of view were imaged at 10,000× magnification per animal. Images were used to count myelinated axons, measure axon diameters, and g-ratios in FIJI. For g-ratio analysis, a minimum of 90 axons per animal from a minimum of three mice per group were measured.

### Primary mouse OPC culture

Oligodendrocyte progenitor cells were isolated from P5 to P6 wild-type or transgenic mouse pups using a previously published protocol (Emery and Dugas, 2013). Cultures were grown on poly-d-lysine (PDL)-coated vessels in defined serum-free media and supplied daily with Platelet-Derived Growth Factor AA (PDGF-AA) (10 ng/ml; PeproTech), Neurotrophin 3 (NT-3; 1 ng/ml, PeproTech), and ciliary neurotrophic factor (CNTF; 10 ng/ml; PeproTech). For the differentiation assay, PDGF is withdrawn from OPC culture, and cells were cultured in Sato media containing OL differentiation factor thyroid hormone T3 (3,3′,5-Triiodo-L-thyronine sodium; 4 ng/ml in Sato media; Sigma-Aldrich), CNTF (10 ng/ml), forskolin (5 µM), and NT-3 (1 ng/ml). For small molecule inhibitor experiments, OPCs were either cultured in the differentiating condition (see above) with LDN-193189 (0.2 µm; Stemgent) or vehicle (DMSO) being added 30 min before BMP4 addition (1 ng/ml; catalog #314-BP, R&D Systems). In some cultures, OPCs were isolated from *Pdgfra-CreER^T2^*::*Bmpr1a* ^fl/fl^ (Cre[+]) and *Bmpr1a* ^fl/fl^ control (Cre[−]) mice. These OPCs were treated with 4-hydroxy-tamoxifen (referred to as “4OHT,” 500 nM in EtOH; Sigma-Aldrich) to induce the knockout of BMPRIA or an equal volume of vehicle (ethanol). For the differentiation assay, OPCs were treated with either BMP4 (1 ng/ml) or vehicle (0.1% BSA in D-PBS) with the addition of differentiation Sato media containing T3. For some experiments, 4OHT or vehicle (ethanol) were added 24 h before BMP4 addition (1 ng/ml; catalog #314-BP, R&D Systems). After a set time point as indicated, cells were fixed in 4% PFA for 20 min followed by immunocytochemical staining (see below). For differentiation assays, three technical replicates and a minimum of three mice per condition or genotype were used.

### Dorsal root ganglion/OPC coculture

Dorsal root ganglion (DRG)/OPC cocultures were established based on published techniques (Xiao et al., 2010). Briefly, OPCs were isolated as detailed above and seeded onto coverslips containing purified DRGs at a density of 2 × 10^5^ OPCs per 22 mm poly-ornithine (Sigma-Aldrich)/PDL-coated coverslip and incubated overnight to facilitate attachment. DRG-OPC cocultures were maintained for 14 d in a defined coculture media containing a 1:1 ratio of Sato medium/Neurobasal medium (Thermo Fisher Scientific) with 2% NeuroCult SM1 supplement (Stem Cell Technologies). Media were changed every 2–3 d. For small-molecule inhibitor experiments, cells were cultured either with LDN-193189 (0.2 µm; Stemgent) or vehicle (DMSO) for 30 min before BMP4 addition (1 ng/ml; catalog #314-BP, R&D Systems) at each feed. For transgenic experiments, OPCs isolated from *Pdgfra-CreER^T2^*::*Bmpr1a* ^fl/fl^ and *Bmpr1a* ^fl/fl^ control (Cre[−]) mice were treated with 4OHT or vehicle control (ethanol) for the first 48 h following coculturing with neurons. After 14 d, cocultures were immunostained or protein was extracted for Western blotting as described below.

### Immunocytochemistry

After fixation with 4% PFA for 18 min, cells were rinsed three times in MT-PBS. Cells were blocked with 10% normal goat serum with 0.3% Triton X-100 in MT-PBS for 60 min at RT, followed an incubation with primary antibodies against GFAP (1:200, mouse, catalog #MAB360, Millipore; 1:200, rabbit, catalog #Z03374, DAKO), MBP (1:50, mouse, catalog #MAB381, Millipore; 1:100, rat, catalog #ab980, Millipore), or rabbit anti-Neurofilament (1:200; catalog #AB1987, Millipore). Cells were then rinsed with MT-PBS followed by the appropriate fluorophore-conjugated secondary antibodies (all 1:500 in antibody diluent, Thermo Fisher Scientific) for 60 min at RT. Cells were rinsed twice in MT-PBS before adding Hoechst (1:10,000 in MT-PBS; catalog #33342, Invitrogen) for 10 min. Cells were rinsed twice in MT-PBS and mounted with Cytomation fluorescence mounting medium (Dako) on SuperFrost Plus glass slides (Thermo Fisher Scientific). Six fields per culture, and three technical replicate from a minimum of three animals per condition or genotype were analyzed, and images captured by a Carl Zeiss Axioplan 2 epifluorescence upright microscope.

### Immunocytochemical quantification

For OPC culture images, all Hoechst^+^ nuclei were counted using Adobe Photoshop (version CS5, Adobe), and the morphology of each Hoechst^+^ cell was designated as an astrocyte (GFAP^+^), immature oligodendrocyte (MBP^+^), or mature oligodendrocyte (MBP^+^), or was unclear (MBP/GFAP^−^). These populations (excluding the “unclear” cells) were then graphed as a proportion of all Hoechst^+^ cells. For the DRG/OPC coculture analysis, an average length for a clearly defined segment was subjectively defined at the start of counting using the ImageJ measure tool, and then the same length is used to count further segments. This was consistently applied throughout all treatments by one counter over one session.

### Western blotting analysis

Total protein of OPC/DRG cocultures was extracted using TNE buffer supplemented with proteinase inhibitor (Roche), separated by SDS-PAGE (200 V; ∼30-40 min) and transferred to PVDF membrane using an iBlot quick transfer dry blot system (Life Technologies). Protein blots were blocked with 5% nonfat milk powder in Tris-buffered saline/Tween 20 (TBST; 50 mm Tris, 150 mm NaCl, 0.05% Tween 20, all from Sigma-Aldrich) for 5-10 min, followed by three rinses with TBST. Blots were subsequently probed with antibodies against myelin proteins MBP (1:50; catalog #AB980, Millipore Bioscience Research Reagents), Myelin oligodendrocyte glycoprotein (MOG) (1:50; catalog #MAB5680, Millipore), or BMPRIA (1:200; catalog #38560, Abcam) overnight at 4°C. An antibody against β-actin (1:5000 in TBST + 2% BSA; catalog #A5441, Sigma-Aldrich) was also added as an internal loading control. Following three rinses with TBST, blots were incubated with HRP-conjugated secondary antibodies (1:5000; Cell Signaling Technology).

### RNA isolation and quantitative real-time PCR analysis

Following differentiation assay, OPCs were rinsed once with cold D-PBS and lysed using a cell scraper with addition of 600 µl RLT-plus buffer (Qiagen) supplemented with 1% 2-mercaptoethanol (Sigma) as an RNase inhibitor. Pure OPC RNA was acquired by following RNeasy Plus Mini protocol (Qiagen). RNA was reverse-transcribed using Applied Biosystems reagents and following manufacturer’s protocol. Following synthesis of cDNA, samples were loaded undiluted into 96-well plates and SYBR Green Master Mix (Applied Biosystems) was added along with primers. The plate was sealed with optical film (Applied Biosystems) and centrifuged for 1 min at 1000 rpm. It was then loaded into an Applied Biosystems ViiA 7 quantitative real-time PCR (qRT-PCR) system. Average expression of housekeeping gene 18S was used to normalize gene expression using the ΔΔCt method. Primer sequences used were shown in Table 1 (all primers are specific for *Mus musculus*).

**Table 1:** Primer sequences used for qRT-PCR

Gene name	Forward primer	Reverse primer
18S	5´CGAACGTCTGCCCTATCAACTT3´	5’ACCCGTGGTCACCATGGTA3’
Myelin basic protein (*Mbp*)	5´CCCGTGGAGCCGTGATC3´	5´TCTTCAAACGAAAAGGGACGAA-3′
Glial fibrillary acidic protein (*Gfap*)	5´CGTTTCTCCTTGTCTCGAATGA3´	5´CCCGGCCAGGGAGAAGT3´
Inhibitor of DNA binding (*Id4*)	5´TTTGCACGTTCACGAGCATT3´	5´GCGGTCATAAAAGAAGAAACGAA3´
Myelin regulatory factor (*Myrf*)	5′AAGGAGCTGCCTATGCTCACCT3′	5′GCCTCTAGCTTCACACCATGCA3′
BMPRIA (*Bmpr1a*)	5´TCATGTTCAAGGGCAGAATCTAGA3´	5´GGCAAGGTATCCTCTGGTGCTA3´
BMPRIB (*Bmpr1b*)	5´GCGCACCCCGATGTTG3´	5´CATGTCCCCTAAGAAGCTTTCTG3´
BMPRIA-ex2	5´GTTCATCATTTCTCATGTTCAAACTA3´	5´AATCAGAGCCTTCATACTTCATACACC3´

### Analyzing multiple transcriptional changes using RT^2^ profiler PCR array

Purified mRNA reverse transcribed using the RT^2^ First Strand kit (catalog #330401, Qiagen) according to the manufacturer instructions. A mouse TGF-β/BMP Signaling Pathway RT^2^ Profiler PCR Array (catalog #PAMM-035C, SABiosciences) was used to assess the expression of 84 genes specific to TGF-β/BMP signaling activity. Reverse-transcribed cDNA was added to SYBR Green ROX Master mix (catalog #330520, Qiagen) as per manufacturer instructions and loaded into the 96-well plate PCR array. Samples were run on an Applied Biosystems ViiA 7 qRT-PCR system (experimental setup settings were provided by SABiosciences). Average transcription of housekeeping genes provided in the PCR array was used to normalize gene expression using the ΔΔCt method. Data were analyzed using an online software program provided by the manufacturer. Data are reported as changes in fold regulation, defined as equal to the fold change when the fold change value is positive, and the negative inverse of the fold change when the fold change value is negative. A full list of genes analyzed using this method can be found at https://www.qiagen.com/us/shop/pcr/primer-sets/rt2-profiler-pcr-arrays/?catno=PAMM-035Z#geneglobe.

### Statistical analysis

All statistical tests were performed using GraphPad Prism 7 (GraphPad Software). Assessors were blinded to conditions, groups, or genotypes during analysis. All data are presented as the mean ± SEM.

## Results

### LDN-193189 infusion promotes remyelination following cuprizone-induced demyelination *in vivo*

To investigate the influence that BMP4/BMPRI signaling exerts on remyelination, we subjected C57BL/6 mice to cuprizone-induced demyelination as published previously (Sabo et al., 2011). Mice were fed cuprizone for 5 weeks to induce demyelination in several white matter tracts of the brain including the CC. Following cuprizone withdrawal, mice were infused with either LDN-193189 (400 ng/d), a previously characterized inhibitor of BMPRIA and BMPRIB receptor signaling (Boergermann et al., 2010), or vehicle (0.1% DMSO in aCSF) for 7 d and allowed to recover. A parallel cohort of control mice were fed cuprizone and killed at the end of a 5 week period (with no recovery) to assess the extent of demyelination.

**Figure 1.**
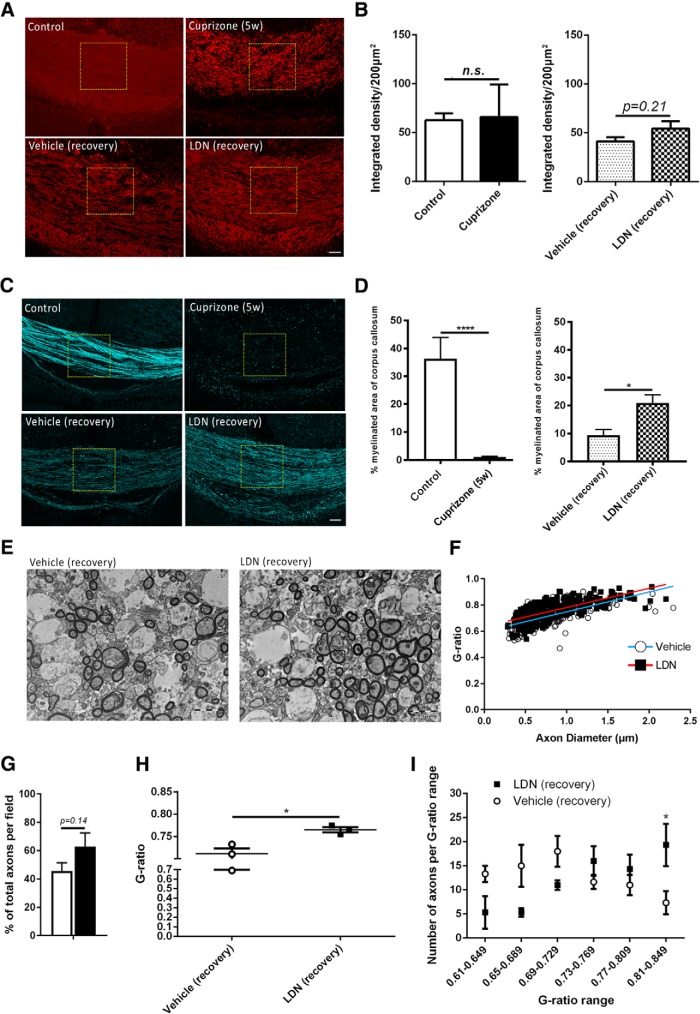
Inhibiting BMP4/BMPRI signaling following demyelination promotes remyelination *in vivo*. ***A***, Representative MBP IHC images showing myelin protein in the caudal corpus callosi of healthy control (control) and 5 week cuprizone-challenged mice (Cuprizone 5w, top panels); and 5 week cuprizone-challenged mice followed by 1 week of recovery with vehicle (Vehicle recovery) or LDN-193189 (LDN recovery) infusion (bottom panels). ***B***, Quantification of integrated density of MBP immunostaining. No significant differences were observed between control and cuprizone-fed mice, or between vehicle- and LDN-infused mice. ***C***, Representative SCoRe images to identify myelin in the caudal corpus callosi of healthy control (control) and 5 week cuprizone-challenged mice (Cuprizone (5w), top panels); and 5 week cuprizone-challenged mice followed by 1 week of recovery with vehicle (Vehicle recovery) or LDN-193189 (LDN recovery) infusion (bottom panels). ***D***, Quantification of myelinated area (SCoRe signal that is pixelated) as a percentage of the total area measured. The SCoRe signal is significantly reduced in 5 week cuprizone-challenged mice [Cuprizone (5w) compared with healthy control (Ctrl) mice, confirming demyelination (***B***)]. LDN-193189-infused mice display a significantly greater SCoRe signal than the vehicle-infused control group (***C***), indicating greater remyelination. ***E***, TEM cross-sectional images of caudal corpus callosum axons of 5 week cuprizone-challenged mice followed by 1 week of recovery with vehicle (Vehicle recovery) or LDN-193189 (LDN recovery) infusion. ***F***, A scatterplot comparison of g-ratio distribution relative to axonal diameter. LDN-infused mice had a significantly higher average g-ratio than vehicle-infused controls (*p* = 0.016). ***G***, Proportion of total myelinated axons in the caudal corpus callosum of vehicle- and LDN-infused mice following 5 weeks of cuprizone administration. A trend, but a nonsignificant increase, was observed in LDN-infused mice compared with vehicle controls. ***H***, The average g-ratio of axons in the caudal corpus callosum of vehicle- and LDN-infused mice following 5 weeks of cuprizone treatment. Mice treated with LDN-193189 after 5 weeks of cuprizone had more thinly myelinated axons (high g-ratio) in the corpus callosum compared with vehicle-infused mice. ***I***, Number of axons in the corresponding g-ratio range for vehicle- versus LDN-infused mice following 5 weeks of cuprizone treatment. EM analysis indicated a higher number of axons with thinner myelin in the LDN-treated group, indicating greater remyelination (*N* = 4-6 animals/group for SCoRe; *N* = 3 animals/group for EM). *p < 0.05, ****p < 0.0001. Scale bars: SCoRe images, 50 µm; TEM images, 2 µm.

The extent of demyelination in the medial caudal corpus callosum of 5-week-old cuprizone-fed mice (no recovery), and the extent of remyelination in cuprizone-fed mice following 7 d infusion with vehicle or LDN-193189 after cuprizone withdrawal was assessed in three ways. We first performed immunohistochemical analysis of the myelin protein marker MBP, as an indicator of myelination. Unchallenged age-matched mice were used as healthy controls to assess the basal level of myelination. While there were clear qualitative effects on MBP staining following cuprizone exposure (Fig. 1*A*, top panels) and LDN infusion (Fig. 1*A*, bottom panels), the assessment of the intensity of MBP staining revealed no significant difference between the groups (Fig. 1*B*). This could be due to the presence of myelin debris (positive for MBP) after cuprizone-induced demyelination. We did observe a trend difference between vehicle- and LDN-infused mice following 1 week of recovery from cuprizone, but this was not significant (Fig. 1*B*, right histogram; *p* = 0.21^a0^; Table 2, statistics). We next used SCoRe imaging to assess the extent of remyelination. SCoRe imaging is a label-free (antibody-free) technique allowing for high-resolution quantitative *in vivo* imaging of substantial areas of myelinated white matter tracts such as the CC (Schain et al., 2014). Using the SCoRe imaging, at the end of 5 weeks of cuprizone feeding, we observed a significant reduction (>10 fold) in the percentage of myelinated area in the corpus callosum of cuprizone-fed mice compared with healthy control mice [Fig. 1*C*, top panels (quantified in *D*); *p* = 0.0047^a^]. When assessing the 1 week recovery groups, we found that mice infused with LDN-193189 for 7 d showed a significant increase (approximately twofold) in the myelinated area compared with vehicle-infused control mice [Fig. 1*C*, bottom panels (quantified in *D*); *p* = 0.014^b^], indicating a greater extent of remyelination.

**Table 2:** Statistics

Test identifier	Type of test	Sample size	Confidence intervals
a0	Student’s unpaired two-tailed *t* test	Three animals per treatment; six technical replicates per animal	−11.1 to 37.1
a	Student’s unpaired two-tailed *t* test	Four control and cuprizone-fed animals, six for vehicle- and LDN-treated animals; three technical replicates per animal	14.81–55.60
b	Student’s unpaired two-tailed *t* test	Four control and cuprizone-fed animals, six for vehicle- and LDN-treated animals; three technical replicates per animal	−20.02 to −2.95
b1	Student’s unpaired two-tailed *t* test	Four vehicle- and five LDN-treated animals; six technical replicates per animal, approximately 100 axons counted per animal	−45.96 to 11.67
b2	Student’s unpaired two-tailed *t* test	Three vehicle- and three LDN-treated animals; six technical replicates per animal, approximately 100 axons counted per animal	0.017–0.091
b3	Two-way ordinary ANOVA with Tukey’s multiple corrections test	Three vehicle- and three LDN-treated animals; six technical replicates per animal, approximately 100 axons counted per animal	−23.39 to −0.6092
b4	Student’s unpaired two-tailed *t* test	Four control and cuprizone-fed animals, six for vehicle- and LDN-treated animals; three technical replicates per animal	0.76–123.91
b5	Student’s unpaired two-tailed *t* test	Four control and cuprizone-fed animals, six for vehicle- and LDN-treated animals; three technical replicates per animal	−81.21 to 26.21
c	Student’s unpaired two-tailed *t* test	Four control and cuprizone-fed animals, six for vehicle- and LDN-treated animals; three technical replicates per animal	−94.40 to −60.46
d	Student’s unpaired two-tailed *t* test	Four control and cuprizone-fed animals, six for vehicle- and LDN-treated animals; three technical replicates per animal	38.05–91.35
e	Student’s unpaired two-tailed *t* test	Four control and cuprizone-fed animals, six for vehicle- and LDN-treated animals; three technical replicates per animal	4.59–17.66
f	Student’s unpaired two-tailed *t* test	Four control and cuprizone-fed animals, six for vehicle- and LDN-treated animals; three technical replicates per animal	−9.19 to −1.36
g	Student’s unpaired two-tailed *t* test	Four control and cuprizone-fed animals, six for vehicle- and LDN-treated animals; three technical replicates per animal	−4980.00 to 2499.00
h	Student’s unpaired two-tailed *t* test	Four control and cuprizone-fed animals, six for vehicle- and LDN-treated animals; three technical replicates per animal	−33,305.00 to −21,828.00
I	Student’s unpaired two-tailed *t* test	Four control and cuprizone-fed animals, six for vehicle- and LDN-treated animals; three technical replicates per animal	−7695.00 to 2665.00
j	Two-way ordinary ANOVA with Tukey’s multiple corrections test	Four independent cultures; three technical replicates per treatment; approximately 500–600 cells counted per treatment	−81.20 to −66.60
k	Two-way ordinary ANOVA with Tukey’s multiple corrections test	Four independent cultures; three technical replicates per treatment; approximately 500–600 cells counted per treatment	−68.00 to −53.30
l	Two-way ordinary ANOVA with Tukey’s multiple corrections test	Four independent cultures; three technical replicates per treatment; approximately 500–600 cells counted per treatment	−23.10 to −8.46
m	One-way ordinary ANOVA with Tukey’s multiple corrections test	Three independent cultures; eight 10× image fields counted per treatment group	19.43–51.24
n	One-way ordinary ANOVA with Tukey’s multiple corrections test	Three independent cultures; eight 10× image fields counted per treatment group	−51.15 to −19.35
o	One-way ordinary ANOVA with Tukey’s multiple corrections test	Three independent cultures; eight 10× image fields counted per treatment group.	−33.99 to −2.18
p	One-way ordinary ANOVA with Tukey’s multiple corrections test	Three independent cultures; three technical replicates per treatment	−5.49 to −2.99
q	One-way ordinary ANOVA with Tukey’s multiple corrections test	Three independent cultures; three technical replicates per treatment	−7.02 to −3.91
r	One-way ordinary ANOVA with Tukey’s multiple corrections test	Three independent cultures; three technical replicates per treatment	1.11–3.61
s	One-way ordinary ANOVA with Tukey’s multiple corrections test	Three independent cultures; three technical replicates per treatment	2.84–5.95
t	One-way ordinary ANOVA with Tukey’s multiple corrections test	Three independent cultures; three technical replicates per treatment	−2.77 to −0.35
u	One-way ordinary ANOVA with Tukey’s multiple corrections test	Three independent cultures; three technical replicates per treatment.	−0.17 to 2.25
v	One-way ordinary ANOVA with Tukey’s multiple corrections test	Three independent cultures; three technical replicates per treatment	−1.36 to −0.16
w	One-way ordinary ANOVA with Tukey’s multiple corrections test	Three independent cultures; three technical replicates per treatment	−2.47 to −0.51
x	Student’s unpaired two-tailed *t* test	Three independent cultures; three technical replicates per treatment	Not available
y	Two-way ordinary ANOVA with Tukey’s multiple corrections test	Three independent cultures; three technical replicates per treatment; approximately 500–600 cells counted per treatment	−89.42 to −43.00
z	Two-way ordinary ANOVA with Tukey’s multiple corrections test	Three independent cultures; three technical replicates per treatment; approximately 500–600 cells counted per treatment	−32.21 to 14.20
aa	Two-way ordinary ANOVA with Tukey’s multiple corrections test	Four independent cultures; three technical replicates per treatment; approximately 500–600 cells counted per treatment	−23.20 to 23.30
ab	Two-way ordinary ANOVA with Tukey’s multiple corrections test	Four independent cultures; three technical replicates per treatment; approximately 500–600 cells counted per treatment	−19.65 to −2.29
ac	Two-way ordinary ANOVA with Tukey’s multiple corrections test	Four independent cultures; three technical replicates per treatment; approximately 500–600 cells counted per treatment	13.07–30.42
ad	Student’s unpaired two-tailed *t* test	Three independent cultures for both Pdgfra-CreER^T2^::Bmpr1a^fl/fl^ and Bmpr1a^fl/fl^ cocultures; eight 10× image fields counted per treatment group	14.65–70.88
ae	Student’s unpaired two-tailed *t* test	Three independent cultures for both PdgfraCreER^T2^::Bmpr1a^fl/fl^ and Bmpr1a^fl/fl^ cocultures; eight 10x image fields counted per treatment group	−39.73 to 36.81

To ascertain the effect of LDN-193189 on the extent of remyelination and ultrastructure of myelinated axons, sagittal sections of caudal corpus callosum were assessed using TEM. Comparing the raw counts of total myelinated axons per image field in the corpus callosum of mice treated with either LDN-193189 or vehicle revealed a trend increase in the percentage of myelinated axons compared with the control group [Fig. 1*E*(quantified in *G*); *p* = 0.20^b1^]. However, when we compared g-ratios (as an indicator of myelin thickness), both the average g-ratio (Fig. 1*H*) and distribution of g-ratios relative to axonal diameter (Fig. 1*F*), were greater in mice infused with LDN-193189 compared with vehicle-infused controls, indicative of thinner myelin (Fig. 1*F*,*H*; *p* = 0.016b2). Thinner myelin sheaths are likely to have been recently myelinated after a demyelinating insult, as they have not completed the full number of wraps around the axon compared with myelin sheaths formed during development (Franklin et al., 2013). Importantly, analysis of the number of myelinated axons grouped by the range of g-ratios demonstrated that the LDN-infused group had significantly more myelinated axons with g-ratios >0.81 (Fig. 1*I*; *p* = 0.035^b3^) compared with the control group, indicative of more axons with thin myelin sheaths. Therefore, our EM results together with the SCoRe imaging data collectively suggest that LDN infusion significantly enhances the extent of remyelination, resulting in more remyelinating axons than the control group.

### LDN-193189 infusion promotes oligodendrocyte differentiation following demyelination *in vivo*

**Figure 2.**
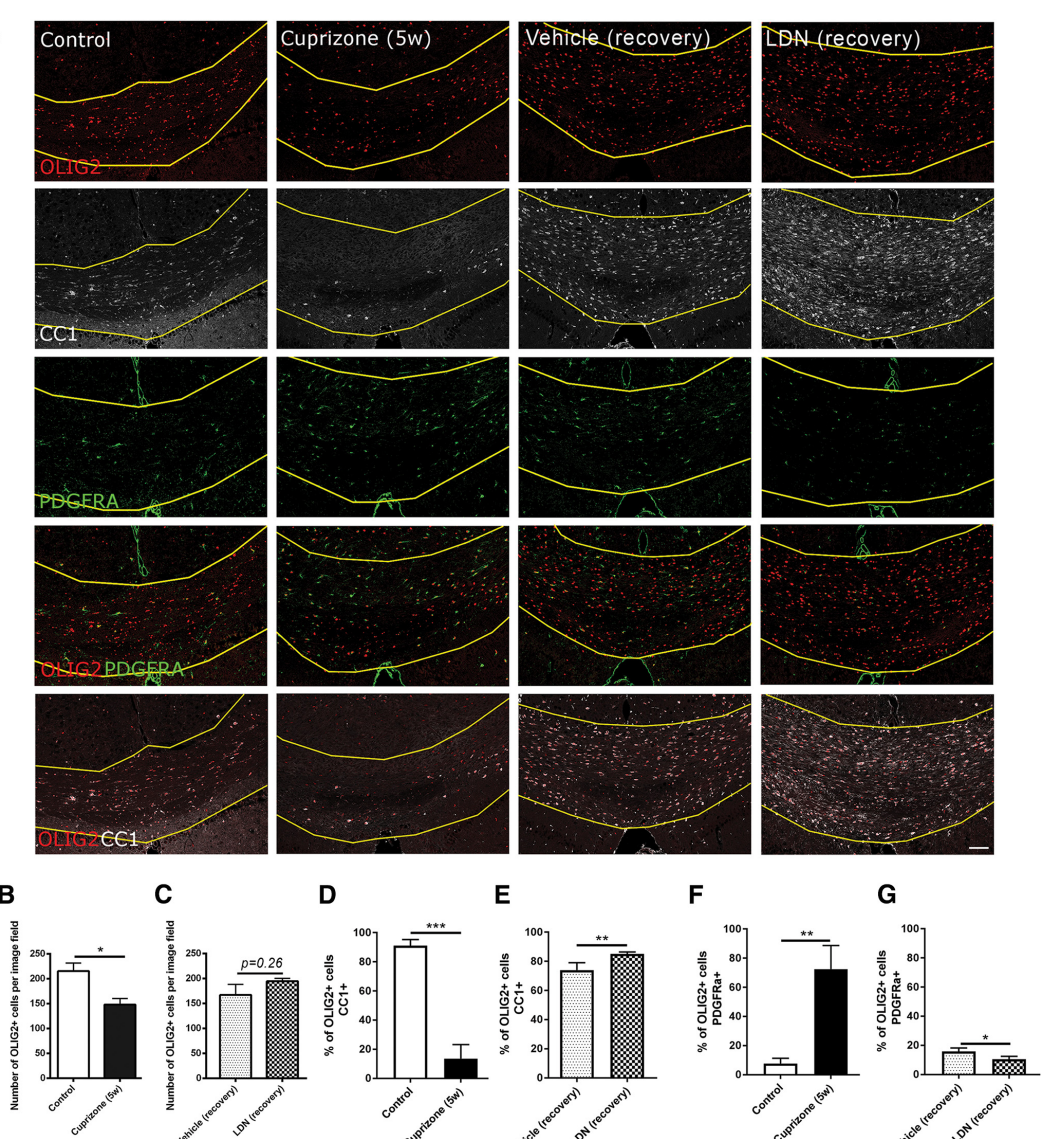
Inhibiting BMP4/BMPRI signaling following demyelination promotes oligodendrocyte differentiation *in vivo*. ***A***, Representative micrographs of immunostaining in the caudal corpus callosi of healthy control mice (control), mice subjected to 5 weeks of cuprizone treatment (Cuprizone 5w), and mice subjected to 5 weeks of cuprizone with either vehicle (Vehicle recovery) or LDN-193189 (LDN recovery) infusion for 1 week, and immunostained with OLIG2 and either PDGFRα or CC1. ***B***, ***C***, Analysis of OLIG2^+^ cell number in healthy control mice (control), mice subjected to 5 weeks of cuprizone treatment (Cuprizone 5w), and mice infused with either vehicle (Vehicle recovery) for 1 week or LDN-193189 (LDN recovery) for 1 week. As expected, the total number of OLIG2^+^ cells is significantly decreased after 5 weeks of cuprizone treatment compared with controls. ***D***, Quantification of the proportion of OLIG2^+^/CC1^+^ mature oligodendrocytes showing a significant reduction at the end of cuprizone feeding. ***E***, LDN-193189-infused mice have a significantly higher proportion of mature oligodendrocytes compared with the vehicle control group following 1 week recovery. ***F***, Quantification of the proportion of OLIG2^+^/PDGFRα^+^ OPCs showing a significant increase at the end of cuprizone feeding. ***G***, LDN-193189-infused mice have a significantly smaller fraction of OPCs compared with the vehicle control group following recovery (*N* = 4-6 animals/group). *p < 0.05, **p < 0.01, ***p < 0.001. Scale bar, 50 µm.

Having shown that infusion of LDN-193189 significantly enhanced the extent of myelin repair *in vivo*, we next sought to determine the effect that infusion exerted on oligodendroglial populations. To address this, we assessed the number of OLIG2^+^ oligodendroglia as well as the proportion of OLIG2^+^/CC1^+^ mature OLs and OLIG2^+^/PDGFRα^+^ OPCs in the medial caudal corpus callosum (Fig. 2). As expected, there was significantly fewer OLIG2^+^ oligodendroglial cells in the corpus callosum of mice treated with cuprizone for 5 weeks versus control mice [Fig. 2*A* (quantified in *B*); *p* = 0.030^b4^]. Interestingly, there was no significant difference in the number of OLIG2^+^ cells between the vehicle- and LDN-treated mice [Fig. 2*A* (quantified in *C*); *p* = 0.26^b5^], suggesting that LDN infusion does alter the overall number of oligodendroglial lineage cells during remyelination. Consistent with previous studies (Keirstead et al., 1998; Chari and Blakemore, 2002), there was a significant reduction in the proportion of OLIG2^+^/CC1^+^ mature OLs at the peak of demyelination (5 weeks of cuprizone) compared with non-cuprizone-challenged healthy control mice [Fig. 2*A* (quantified in *D*); *p* = 0.0002^c^], which is accompanied by a significantly higher proportion of OLIG2^+^/PDGFRα^+^ OPCs [Fig. 2*A* (quantified in *F*); *p* = 0.0025^d^]. Interestingly, after 1 week of recovery following cuprizone withdrawal, LDN-193189-infused mice had a significantly higher proportion of OLIG2^+^/CC1^+^ mature OLs compared with the vehicle infused mice [Fig. 2 (quantified in *E*); *p* = 0.0059^e^]. This is accompanied by fewer OPCs in these animals compared with the vehicle control group [Fig. 2*A* (quantified in *G*); *p* = 0.016^f^]. Thus, our results show that blocking BMP4/BMPRI signaling enhances the differentiation of OPCs into mature OLs during remyelination *in vivo*. Coupled with the SCoRe and TEM analysis, it suggests that inhibiting BMPRIA/B signaling with LDN-193189 leads to a greater number of OPCs contacting axons, differentiating, and forming new myelin; this subsequently leads to a greater number of axons with high g-ratios, indicative of remyelination.

**Figure 3.**
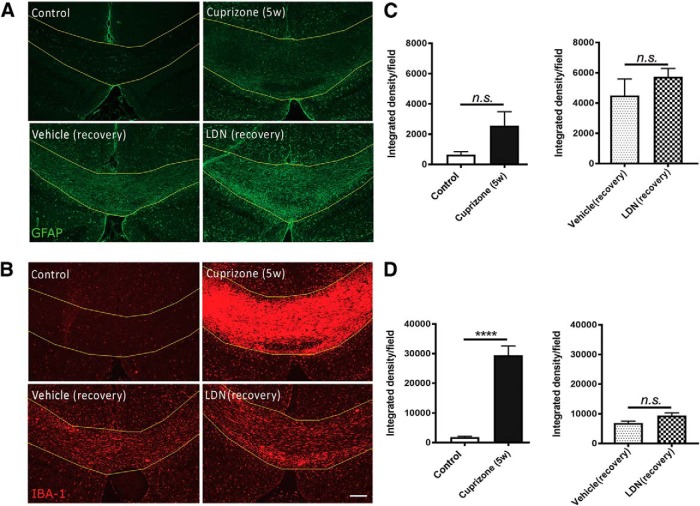
Inhibiting BMP4/BMPRI signaling exerts no influence on astrocytes or microglia *in vivo.* ***A***, ***B***, Representative micrographs of immunostaining in the caudal corpus callosi of healthy control mice (control), mice subjected to 5 weeks of cuprizone treatment (Cuprizone 5w), and mice subjected to 5 weeks of cuprizone treatment with either vehicle (Vehicle recovery) or LDN-193189 (LDN recovery) infusion for 1 week, and immunostained with GFAP (***A***) or IBA-1 (***B***). ***C***, Quantification of the integrated density of GFAP immunofluorescence. There is no significant change in GFAP immunofluorescence at peak demyelination (Cuprizone 5w; left) or following the infusion of LDN-193189 (LDN recovery) for 1 week compared with control groups (Control, Vehicle; right panel). ***D***, Quantification of the integrated density of IBA-1 immunofluorescence. There is a significant increase in IBA-1 immunofluorescence in the corpus callosum at peak demyelination (Cuprizone 5w; left); however, there is no significantly different increase in IBA-1 immunofluorescence between vehicle (Vehicle recovery) or LDN-193189 (LDN recovery) infusion during 1 week of recovery after cuprizone treatment (right; *N* = 4-6 animals/group). ****p < 0.0001. Scale bar, 50 µm.

It has been previously identified that exogenous BMP4 promotes astrogliogenic effect *in vitro* and *in vivo,* whereas blocking its signaling inhibits this effect (Grinspan et al., 2000; See et al., 2004; Sabo et al., 2011). Thus, we next investigated whether LDN-193189 infusion also affected astrocytes (Fig. 3*A*,*C*). Immunostaining of caudal corpus callosum sections of normal control mice for GFAP showed a low level of positive immunostaining. As astrocytes can both proliferate and ramify in response to injury (Williams et al., 2007), we assessed the integrated density of GFAP fluorescence of the section and observed a substantial increase in GFAP immunofluorescence signal at the end of 5 weeks of cuprizone feeding compared with healthy controls [Fig. 3*A* (quantified in *C*)]. This is expected, as astrogliosis is observed from 3 to 4 weeks after cuprizone (Hibbits et al., 2012). Interestingly, the administration of LDN-193189 resulted in no significant effect on GFAP immunofluorescence intensity compared with the vehicle control [Fig. 3*A* (quantified in *C*);^g^ *p* = 0.85], suggesting that blocking BMP4/BMPRI signaling via LDN-193189 infusion exerted little influence on astrogliosis during remyelination *in vivo*.

As microglia represent a considerable proportion of cells in the corpus callosum during cuprizone-induced demyelination (Gudi et al., 2014), we then assessed whether LDN-193189 infusion affected microglia by quantifying the degree of IBA^+^ immunofluorescence in the corpus callosum (Fig. 3*B*,*D*). As expected, there is a significant increase in the integrated density of IBA-1 immunofluorescence in the caudal corpus callosum of mice at the peak of demyelination (following 5 weeks of cuprizone) compared with healthy controls [Fig. 3*B*, (quantified in *D*); *p* < 0.0001^h^], indicating a dramatic increase in the inflammatory response to demyelination. However, there was no significant difference in the integrated density of IBA-1 immunofluorescent between mice infused with LDN-193189 and vehicle following 1 week of recovery [Fig. 3*B* (quantified in *D*); *p* = 0.61^i^], suggesting that LDN-193189 exerted no significant influence on microglia during remyelination *in vivo.* Together, these data suggest that blocking BMP4/BMPRI signaling in the murine cuprizone model of demyelination exerts little effect on either astrocytes or microglia, but rather selectively enhances OPC differentiation to promote myelin repair *in vivo*.

### Inhibiting BMP4/BMPRI signaling promotes oligodendroglial differentiation and myelination *in vitro*

**Figure 4.**
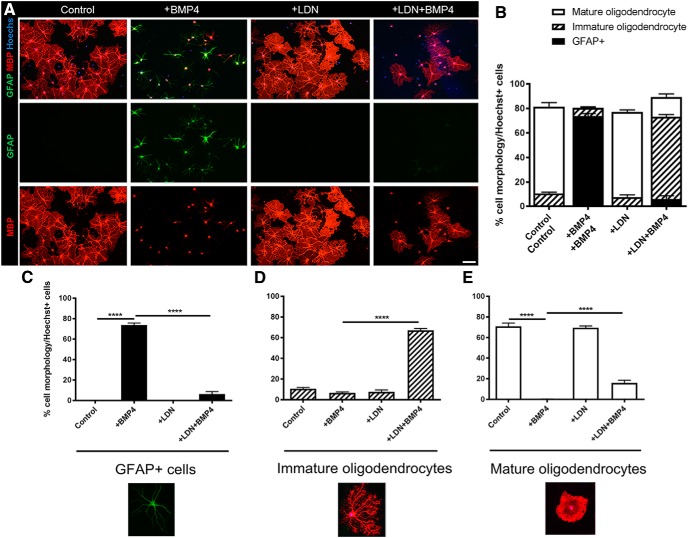
BMP4 signals via BMPR1 in OPCs to enhance oligodendrocyte differentiation and reduce astrogliogenesis *in vitro.* ***A***, Representative micrographs of immunostaining of differentiated OPC cultures for MBP and GFAP under untreated (Control) conditions, or following treatment with BMP4, LDN-193189 (LDN), or both BMP4 and LDN-193189 (LDN^+^BMP4). ***B***, Quantification of cell phenotypic distribution for each condition based on GFAP expression and MBP^+^ morphology. MBP^+^ cells were classified as either mature (flattening of branched extracellular membrane) or immature (branched morphology but not fused layers). ***C***, Quantification of the proportion of GFAP^+^ cells in the cultures. BMP4 significantly increased the proportion of GFAP^+^ cells compared with untreated (Control) cultures. While LDN-193189 (LDN) alone exerted no significant effect, pretreatment with LDN before BMP4 (LDN^+^BMP4) significantly abrogated effect of BMP4 on astrocytes. ***D***, Quantification of the proportion of immature oligodendrocytes in the cultures. Treatment with BMP4 or LDN-193189 (LDN) exerted no significant effect, whereas pretreatment with LDN-193189 before BMP4 (LDN^+^BMP4) significantly increased the proportion of immature oligodendrocytes. ***E***, Quantification of the proportion of mature oligodendrocytes in the cultures. Treatment with BMP4 significantly blocked OPC differentiation, whereas LDN-193189 (LDN) alone exerted no significant effect. Pretreatment with LDN before BMP4 (LDN^+^BMP4) significantly abrogated the effect of BMP4 on oligodendrocyte differentiation (*N* = 4 animals/group). ****p < 0.0001. Scale bar, 20 µm.

The *in vivo* data suggest that LDN-193189 is exerting its effects selectively on OPC differentiation to promote remyelination. To further establish whether LDN-193189 mediates its promyelinating effect directly on oligodendroglia, we used *in vitro* OPC monocultures and myelinating cocultures to examine the effect of BMP4 and LDN-193189 on OPC differentiation and myelination, respectively. To assess differentiation, isolated primary mouse OPCs were exposed to T3 to initiate differentiation, in the presence of either LDN-193189, BMP4, both (LDN^+^BMP4, with BMP4 being added after 30 min after LDN-193189), or vehicle for 72 h (Fig. 4). The majority (∼70%) of vehicle-treated OPCs differentiated into MBP^+^ mature oligodendrocytes [Fig. 4*A* (quantified in *B*,*E*)], characterized by a flat morphology as the cells extended their developing myelin sheath across the 2D surface of the coverslip. This contrasted with the immature phenotype, where the processes of differentiating oligodendrocytes have extended, but have not begun spreading out and fusing. Concordant with previous studies (Mabie et al., 1997; Grinspan et al., 2000), OPCs treated with BMP4 primarily (∼70%) differentiated into GFAP^+^ astrocytes compared with vehicle control OPC cultures [Fig. 4*A* (quantified in *B*,*C*); *p* < 0.0001^j^]. While LDN-193189 treatment alone did not significantly influence OPC differentiation at the basal level, it significantly blocked the astrogliogenic effect that BMP4 exerted on the OPC cultures, as evidenced by significantly more oligodendrocytes (both immature and mature phenotypes) in LDN plus BMP4-treated cultures than BMP4 alone cultures [Fig. 4*A* (quantified in *B*,*D*,*E*); *p* < 0.0001^k,l^]. These data demonstrate that blocking BMPRI signaling in OPCs reduces the astrogliogenic effect of BMP4 and promotes the differentiation of OPCs into mature oligodendrocytes, suggesting that BMP4 signals via BMPRI receptors in OPCs to exert an inhibitory effect on oligodendrocyte differentiation.

**Figure 5.**
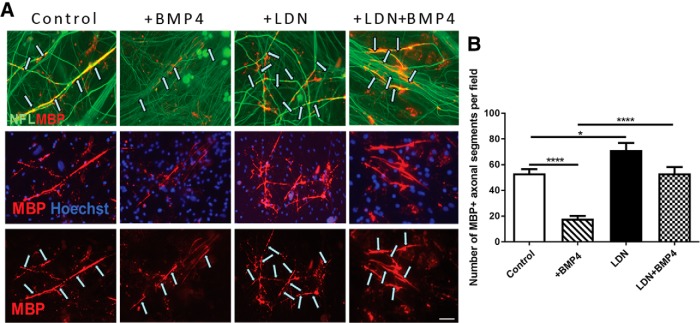
BMP4 signals via BMPR1 in OPCs to promote myelin formation *in vitro*. ***A***, Representative micrographs of myelinating DRG/OPC cocultures treated with vehicle (control), BMP4, LDN-193189 (LDN), or both LDN-193189 and BMP4 (LDN+BMP4) for 14 d and immunostained for MBP and Neurofilament. Arrows indicate MBP^+^ myelin segments colabeled with NFL^+^ axons. ***B***, Quantification of the number of MBP^+^ myelinated axonal segments per field from these cocultures. BMP4 treatment significantly reduced the number of MBP^+^ myelin segments compared with cocultures, which is blocked by the pretreatment of LDN-193189 (LDN+BMP4; *N* = 4 independent cocultures/group). *p < 0.05, ****p < 0.0001. Scale bar, 30 µm.

We next assessed whether the effect that LDN-193189 exerts on potentiating OL differentiation also enhances myelination using the well established DRG neuron/OPC myelinating coculture assay (Xiao et al., 2010; Fig. 5). Consistent with a previous report (See et al., 2004), there is significantly fewer MBP^+^ myelinated axonal segments (approximately threefold reduction) in exogenous BMP4-treated cocultures compared with vehicle-treated control cocultures (Fig. 5*A*,*B*; *p* < 0.0001^m^), suggesting that BMP4 inhibits myelination *in vitro*. Importantly, this BMP4-induced inhibitory effect on myelination is blocked by pretreatment with LDN-193189 before BMP4 exposure (Fig. 5*A*,*B*; *p* < 0.0001^n^), suggesting that BMP4 signals via BMPRI to exert this inhibitory effect. Interestingly, LDN-193189 treatment alone also resulted in a significant increase in the number of myelinated segments compared with baseline vehicle controls (Fig. 5*A*,*B*; *p* = 0.019^°^), suggesting there is some endogenous BMP4 present in the cocultures. Together, our results suggest that BMP4 signals via BMPRI in OPCs to inhibit their differentiation into mature OL and subsequent myelination.

### Inhibiting BMP4/BMPRI signaling in OPCs alters the expression of the transcriptional repressor Id4

**Figure 6.**
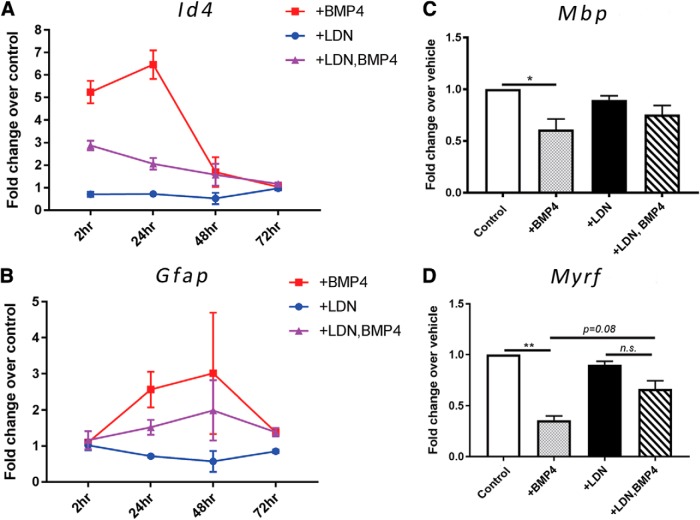
Inhibiting BMP4/BMPRI signaling in OPCs alters the expression of transcription factor *Id4* and *Gfap*, but not *Mbp* or *Myrf*. ***A***, ***B***, qRT-PCR analysis of *Id4* and *Gfap* transcript levels from OPCs cultured in differentiation media and treated with LDN-193189, BMP4, or both, or vehicle (control) over various time points. *BMP4 significantly increased the level of *Id4* transcripts at 2 and 24 h compared with the control, and this upregulation is blocked by pretreatment with LDN-193189 before BMP4 exposure. *Gfap expression was also significantly reduced by pretreatment of OPCs with LDN-193189 before BMP4 exposure. ***C***, ***D***, qRT-PCR analysis of myelin protein gene *Mbp* and key myelination transcription factor *Myrf* from OPCs treated with LDN-193189, BMP4, or both, or vehicle over 24 h. BMP4 significantly reduced the expression level of both *Mbp* and *Myrf* genes, with this effect reduced by LDN-193189 pretreatment; *N* = 3 independent cultures/group). *p < 0.05, **p < 0.01.

Previous research strongly suggests that BMP4 inhibits the differentiation of oligodendrocyte-lineage cells by upregulating Id4, a transcription factor that inhibits OL differentiation (Samanta and Kessler, 2004). To understand whether the effect observed on OPC differentiation and myelination was mediated, at least partially, by Id4, we used qRT-PCR to examine changes in transcription levels of *Id4* as well as *Gfap*, *Mbp*, and myelin regulatory factor (*Myrf*) in OPCs treated with BMP4 and/or LDN-193189. To do this, we repeated the differentiation assay in OPC monocultures in the presence or absence of LDN-193189 and BMP4 over various time points (Fig. 6). We found there was a significant increase in the level of *Id4* transcription in BMP4-treated OPCs compared with control untreated cultures at 2 h (approximately fivefold; Fig. 6*A*; *p* < 0.0001^p^), which peaked at 24 h (approximately sixfold; Fig. 6*A*; *p* < 0.0001^q^). Interestingly, this BMP4-induced increase in *Id4* transcription is abolished by pretreatment with LDN-193189 at both the 2 and 24 h time points [Fig. 6*A*; *p* = 0.0014^r^ (2 h); *p* < 0.0001^s^ (24 h)]. BMP4 treatment also led to a significant increase in *Gfap* transcription at 24 h (Fig. 6*B*; *p* = 0.014^t^), which was attenuated by the pretreatment with LDN-193189, but not significantly (Fig. 6*B*; *p* = 0.094^u^). Further, BMP4 treatment significantly reduced the expression of *Mbp* and *Myrf* transcripts at the 24 h mark compared with vehicle treated cultures [Fig. 6*C*,*D*; *p* = 0.016^v^ (*Mbp*), *p* = 0.0053^w^ (*Myrf*)]. Collectively, these data suggest that BMP4 signals to BMPRI in OPCs to upregulate *Id4*, coinciding with an increase in *Gfap* transcription and downregulation of myelin genes *Mbp* and *Myrf*.

We further explored the downstream transcriptional effects that BMP4 and LDN-193189 exerted on OPCs using the RT2 PCR Profiler Array Kit measuring the transcription of 84 genes related to the TGF-β/BMP signaling family. To address this, OPC monocultures were treated with either LDN-193189, BMP4, both (LDN^+^BMP4) or vehicle and allowed to differentiate for 24 h before RNA analysis. We compared changes in transcription within the following three comparisons: (1) control OPCs versus BMP4-treated OPCs; (2) control OPCs versus LDN-193189-treated OPCs; and (3) BMP4-treated OPCs versus LDN-193189^+^BMP4-treated OPCs. A summary of genes with a significant fold regulation of greater than two is presented in Table 3 ^x^. We found that BMP4-treated OPCs significantly increased the transcription of several TGF-β target genes, as well as *Id1* and *Id2*. Interestingly, the genes of several BMP signaling regulatory proteins such as noggin, BAMBI, and BMP binding endothelial regulator (BMPER) were also upregulated, suggesting the possibility that exogenous BMP4 treatment of OPCs also activates intrinsic self-feedback mechanisms to modify the levels of BMP4 signaling. The transcription of *Bmp4* itself was downregulated by exogenous BMP4 treatment in OPCs. Interestingly, BMP4 treatment significantly upregulates *Smad1* but not *Smad5*; this is reversed in OPC cultures pretreated with LDN-193189 before BMP4 exposure. *Smad2*, which is not typically used by BMP4 (Miyazono et al., 2010), was also downregulated, suggesting that *Smad5* may also be similarly unused by BMP4 in OPCs.

**Table 3:** Summary of differentially regulated BMP/TGF-β signaling pathway genes in OPCs cultured in LDN-193189, BMP4, or both, or vehicle for 24 h in differentiating conditions

	Up/down	Fold regulation	*p* value
BMP4 vs control			
Gene name			
Epithelial membrane protein 1 (*Emp1*)	↑	9.19	*
Noggin (*Nog*)	↑	6.20	***
Growth arrest and DNA-damage-inducible 45 β (*Gadd45b*)	↑	5.17	*
Cyclin-dependent kinase inhibitor 1A (*Cdkn1a*)	↑	5.11	**
Transforming growth factor, beta 3 (*Tgfb3*)	↑	4.37	*
Jun-B oncogene (*Junb*)	↑	4.27	*
Latent transforming growth factor beta binding protein 1 (*Ltbp1*)	↑	3.94	**
BMP and activin membrane-bound inhibitor (*Bambi*)	↑	3.84	**
BMP-binding endothelial regulator (*Bmper*)	↑	3.40	**
Inhibitor of DNA binding 2 (*Id2*)	↑	2.25	*
Distal-less homeobox 2 (*Dlx2*)	↑	2.10	*
TGFβ-1-induced transcript (*Tgfb1i1*)	↑	2.06	*
Inhibitor of DNA binding 1 (*Id1*)	↑	1.94	*
FBJ osteosarcoma oncogene (*Fos*)	↑	1.94	**
SRY-box containing gene 4 (*Sox4*)	↑	1.87	*
Small MAD homolog 1 (*Smad1*)	↑	1.50	*
BMP receptor 1A (*Bmpr1a*)	↑	1.54	*
Small MAD homolog 5 (*Smad5*)	↓	−1.27	*
Signal transducer and activator of transcription (*Stat1*)	↓	−1.35	*
TGF-β receptor I (*Tgfbr1*)	↓	−1.60	*
Small MAD homolog 2 (*Smad2*)	↓	−1.66	**
Small MAD homolog 7 (*Smad7*)	↓	−1.74	*
SMAD specific E3 ubiquitin protein ligase 1 (*Smurf1*)	↓	−2.13	****
Plasminogen activator, urokinase (*Plau*)	↓	−5.16	**
Bone morphogenetic protein 4 (*Bmp4*)	↓	−5.53	*
			
LDN-193189 vs control^a^			
Gene name			
Epithelial membrane protein 1 (*Emp1*)	↓	−2.07	*
Inhibitor of DNA binding 2 (*Id2*)	↓	−3.15	**
BMP-binding endothelial regulator (*Bmper*)	↓	−3.63	**
Inhibitor of DNA binding 1 (*Id1*)	↓	−3.65	*
Noggin (*Nog*)	↓	−6.35	*
MDS1 and EVI1 complex locus (*Mecom*)	↓	−22.15	**
			
LDN-193189+BMP4 vs BMP4^b^			
Gene name			
Bone morphogenetic protein 4 (*Bmp4*)	↑	3.32	*
TGF-β receptor I (*Tgfbr1*)	↑	1.69	*
Small MAD homolog 5 (*Smad5*)	↑	1.58	*
Signal transducer and activator of transcription (*Stat1*)	↑	1.41	*
Noggin (*Nog*)	↓	−1.36	*
Distal-less homeobox 2 (*Dlx2*)	↓	−1.40	*
FBJ osteosarcoma oncogene (*Fos*)	↓	−1.42	*
*Col1a1*	↓	−1.64	*
BMP-binding endothelial regulator (*Bmper*)	↓	−2.12	*
BMP and activin membrane-bound inhibitor (*Bambi*)	↓	−2.22	*
Transforming growth factor beta-1-induced transcript 1 (*Tgfb1i1*)	↓	−2.50	*
Cyclin-dependent kinase inhibitor 1A (*Cdkn1a*)	↓	−3.23	**
Insulin-like growth factor 1 (*Igf1*)	↓	−3.50	**
Jun-B oncogene (*Jun*)	↓	−4.19	*
Latent transforming growth factor beta binding protein 1 (*Ltbp1*)	↓	−5.46	***
Epithelial membrane protein 1 (*Emp1*)	↓	−9.63	*

*^a^*Values are compared with controls.

*^b^*Values are compared with BMP4.

*p < 0.05, **p < 0.01, ***p < 0.001, ****p < 0.0001.

Furthermore, we found that OPCs treated with LDN-193189 significantly downregulated *Id1* and *Id2*, as well as levels of the BMP antagonist noggin. Pretreatment of OPCs with LDN-193189 before BMP4 exposure reversed the transcriptional levels of several genes differentially regulated by BMP4 treatment, including *Bmper*, *Bambi*, and *Emp1.* Levels of *Id1* and *Id2* were not significantly downregulated as a result of LDN-193189 pretreatment, in contrast with decreased *Id4* transcription in OPC cultures pretreated with LDN-193189 before BMP4 exposure (identified by an individual *Id4* qRT-PCR). Together, the data suggest that BMP4 inhibits OPC differentiation and their subsequent capacity to myelinate axons via signaling through BMPR1 and regulating an array of downstream signaling molecules and transcription factors in OPCs.

### Deleting OPC-expressed BMPRIA receptors promotes differentiation and myelination *in vitro*

**Figure 7.**
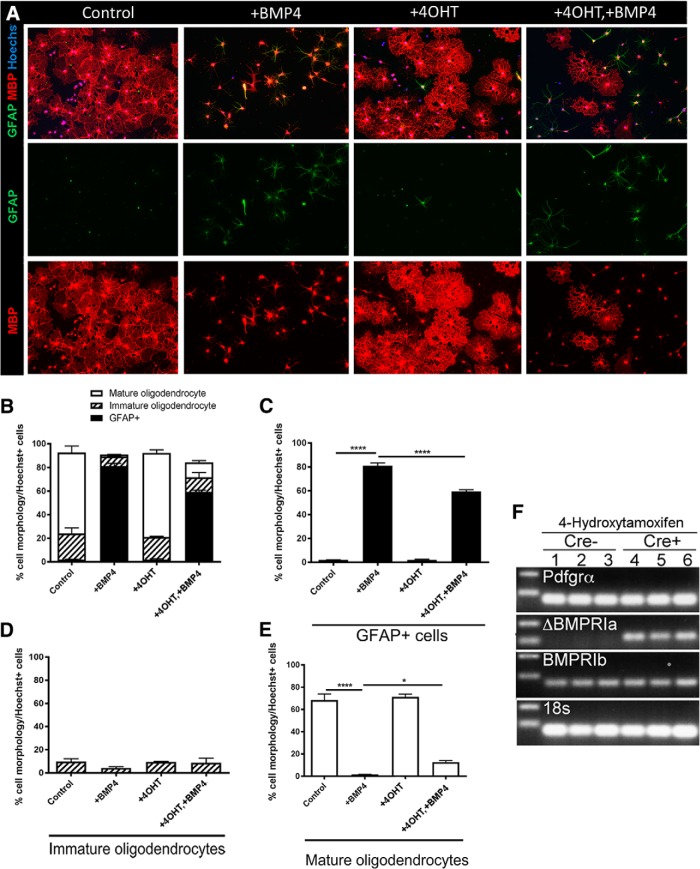
BMP4 signals via BMPR1A in OPCs to potentiate oligodendrocyte differentiation and reduce astrogliogenesis *in vitro.* ***A***, Representative micrographs of immunostaining of differentiated OPC cultures (isolated from *Pdgfra-CreER^T2^*::*Bmpr1a* ^fl/fl^ mice) for MBP and GFAP under untreated (Control) conditions, or following treatment with BMP4, 4OHT, or both BMP4 and 4OHT (^+^4OHT^+^BMP4). ***B***, Quantification of cell-phenotypic distribution for each condition based on GFAP expression and MBP^+^ morphology, as described above (Fig. 4*B*). ***C***, Quantification of the proportion of GFAP^+^ cells in the cultures. BMP4 significantly increased the proportion of GFAP^+^ cells compared with untreated (Control) cultures, whereas 4OHT alone exerted no significant effect. Pretreatment with 4OHT before BMP4 (^+^4OHT^+^BMP4) significantly attenuated the effect of BMP4. ***D***, Quantification of the proportion of immature oligodendrocytes in the cultures. Treatment with BMP4, 4OHT, or both BMP4 and 4OHT (^+^4OHT^+^BMP4) exerted no significant effect. ***E***, Quantification of the proportion of mature oligodendrocytes in the cultures. Treatment with BMP4 significantly decreased OPC differentiation, whereas 4OHT alone exerted no significant effect. Pretreatment with 4OHT before BMP4 (^+^4OHT^+^BMP4) significantly attenuated the inhibitory effect of BMP4 on OPC differentiation. ***F***, PCR analysis of 4OHT-treated OPCs to assess *Bmpr1a* knockout. *Pdgfra-CreER^T2^*::*Bmpr1a* ^fl/fl^ and Cre[−] OPCs were isolated and treated with 4OHT for 24 h and analyzed for the transcription of a sequence corresponding to *Bmpr1a*-*ex2*, rendering the resulting protein untranscribable; *N* = 4 animals/group). *p < 0.05, ****p < 0.0001. Scale bar, 20 µm.

LDN-193189 is known to disrupt BMP4 signaling by inhibiting both BMPRIA and BMPRIB, and, while mouse OPCs express both BMPRIA and BMPRIB, BMPRIA is expressed at a substantially higher level than BMPRIB (Zhang et al., 2014). Thus, it remains unclear whether the aforementioned effect of LDN-193189 on OPC differentiation and myelination are mediated via BMPRIA, BMPRIB, or both. Further, it also remained possible that BMP4 signaling in neurons may influence myelination in the coculture setting. To unequivocally determine whether BMP4 selectively signals to BMPRIA in OPCs to regulate their differentiation and myelination, we adopted a genetic approach, specifically deleting BMPRIA from OPCs. The BMPRIA KO mice are embryonic lethal: thus, we generated *Pdgfra-CreER^T2^*::*Bmpr1a* ^fl/fl^ conditional KO mice, allowing 4OHT-dependent Bmpr1a deletion in *Pdgfra-*expressing OPCs. We first confirmed 4OHT-mediated knockout of *Bmpr1a* in primary OPCs using PCR. OPCs were isolated from *Pdgfra-CreER^T2^*::*Bmpr1a* ^fl/fl^ (Cre[+]) and *Bmpr1a* ^fl/fl^ control (Cre[−]) mice, treated with 4OHT followed by RNA extraction. PCR analysis confirmed the expression of Cre-recombinase in the 4OHT-treated cells, as well as deletion of exon 2 of the Bmpr1a sequence (Fig. 7*F*, ΔBMPRIa panel), while Bmpr1b transcription was unaffected. Analysis of 18S confirmed similar levels of RNA were analyzed (Fig. 7*F*, 18s panel).

To investigate the effect that BMPRIA signaling exerts on OPC differentiation, cells were isolated from Cre[+] and Cre[−] control mice and exposed to 4OHT or vehicle for 24 h, followed by a 72 h differentiation assay in the presence or absence of BMP4. Cultures were assessed for the proportion of postmitotic OLs and astrocytes via immunostaining for MBP and GFAP, respectively (Fig. 7*A*). Consistent with previous results (Fig. 4), in the control condition, the majority (>60%) of OPCs differentiated into mature myelinating OLs after 72 h at the basal level [Fig. 7*A* (quantified in *B*,*D*,*E*)]. As expected, exogenous BMP4 significantly inhibited OPC differentiation compared with the vehicle control, with the vast majority (∼80%) of cells being GFAP^+^ astrocytes in BMP4 alone-treated cultures after 72 h [Fig. 7*A* (quantified in *B*,*C*); *p* < 0.0001^y^]. Treatment with 4OHT exerted no effect on the proportion of OLs [Fig. 7*A* (quantified in *B*,*D*,*E*); *p* = 0.711^z^] or astrocytes [Fig. 7*A* (quantified in *B*,*C*); *p* > 0.999^aa^], but, importantly, it resulted in significantly more OL differentiation and less astrogliogenesis following BMP4 treatment (Fig. 7*E*; *p* = 0.011^ab^), potentiating astrogliosis (Fig. 7*B*,*D*,*E*; *p* < 0.0001^ac^). These results collectively suggest that BMP4 signals via BMPRIA within OPCs to inhibit their differentiation.

**Figure 8.**
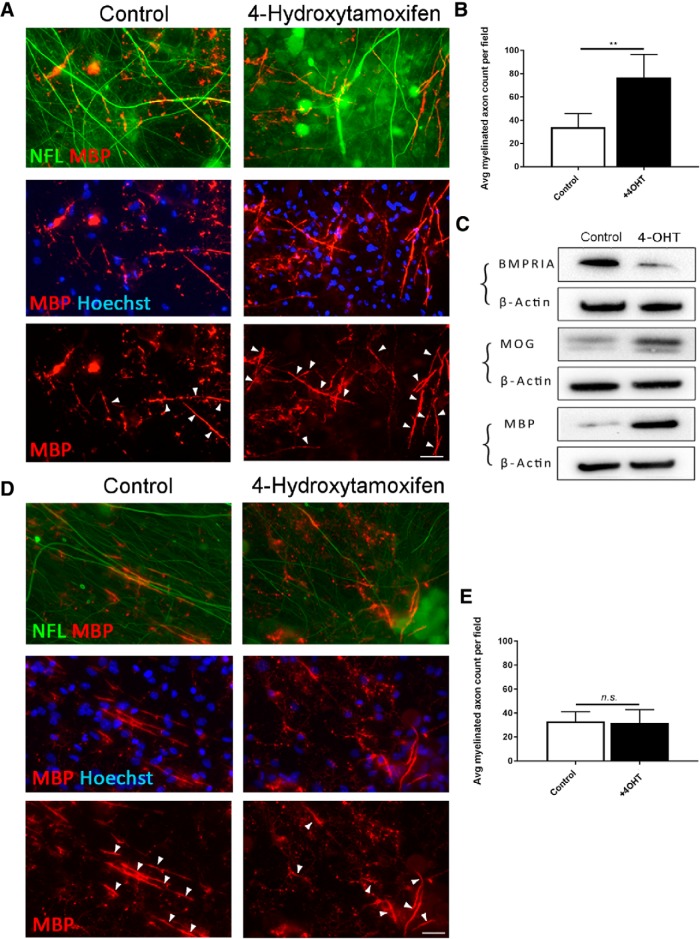
BMP4 signals via BMPR1A in OPCs to promote myelination *in vitro*. ***A***, Representative micrographs of immunostaining for MBP and Neurofilament (NFL) in myelinating cocultures containing OPCs isolated from *Pdgfra-CreER^T2^*::*Bmpr1a* ^fl/fl^ mice. The cocultures were treated with or without 4OHT for 24 h before 14 d of myelination. Arrows indicate MBP^+^ myelinated axons segments colabeled with NFL^+^ axons. ***B***, Quantification of MBP^+^ myelinated axonal segments from these cocultures. 4OHT-induced BMPRIA ablation in OPCs causes significantly more MBP^+^ myelinated axonal segments compared with controls. ***C***, Western blot analysis of BMPRIA and myelin proteins (MOG and MBP) in sister cocultures from ***A*** and ***B***, treated with either 4OHT or vehicle. Treatment with 4OHT substantially reduced BMPRIA expression and leads to qualitatively more myelin proteins (MBP and MOG) expression compared with controls. ***D***, Representative micrographs of immunostaining for MBP and NFL in myelinating cocultures containing OPCs isolated from *Bmpr1a* ^fl/fl^ (Cre^−^ control) mice. The cocultures were treated with or without 4OHT for 24 h before 14 d of myelination. Arrows indicate MBP^+^ myelin segments colabeled with NFL^+^ axons. ***E***, Quantification of MBP^+^ myelinated axonal segments from cocultures. Treatment with 4OHT did not exert a significant effect on myelination in the Cre^−^ cocultures (*N* = 3 independent cultures/treatment group). **p < 0.01. Scale bar, 30 µm.

To investigate whether BMPRIA also mediates the subsequent capacity to myelinate axons, we repeated the myelinating cocultures containing OPCs isolated from Cre[+] and Cre[−] control mice. Cocultures were exposed to 4OHT or vehicle for 24 h and maintained for 14 d followed by immunocytochemical and biochemical analyses of myelination *in vitro.* We found that, in cocultures containing BMPRIA-null OPCs (isolated from Cre[+] mice), 4OHT treatment resulted in significantly more MBP^+^ myelinated axonal segments compared with vehicle-treated control cultures (Fig. 8*A*,*B*; *p* = 0.0098^ad^). Concordant with this, Western blot analysis of myelin proteins MBP and MOG shows that there was qualitatively more myelin protein expression in 4OHT-treated cocultures compared with vehicle controls (Fig. 8*C*). In contrast, 4OHT exerted no effect on myelin formation in cocultures containing OPCs from *Bmpr1a*^fl/fl^ control (Cre[−]) mice (Fig. 8*D*,*E*; *p* = 0.92^ae^). Together, our data suggest that selectively blocking BMP4 signaling in OPCs through ablating BMPRIA promotes oligodendroglial differentiation, reduces astrogliogenesis, and leads to a greater extent of myelination *in vitro*, indicating that BMP4 selectively signals via BMPRIA in OPCs to block oligodendroglial differentiation and myelination.

## Discussion

Identifying the mechanisms that inhibit OL differentiation and remyelination is crucial for developing future strategies that directly target myelin repair in MS. Here we have identified that inhibiting BMP4/BMPRI signaling following cuprizone-induced central demyelination significantly enhances oligodendroglial differentiation and promotes myelin repair in the brain *in vivo*. We have further determined that BMP4 signals to BMPRIA receptors in OPCs to inhibit OL differentiation and myelination *in vitro*. Together, the results of this study identify that inhibiting BMP4/BMPRIA signaling within OPCs promotes CNS remyelination via potentiating OL differentiation, and that blocking this pathway within OPCs is a potential strategy to enhance remyelination.

### Disrupting BMP4/BMPR1 signaling promotes remyelination via potentiating oligodendrocyte differentiation *in vivo*

The results of this study strongly support a role for blocking BMP4/BMPR1 receptor signaling in promoting CNS remyelination. BMP4/BMPRI signaling is upregulated during the remyelinating phase after myelin injury (Cate et al., 2010), and blocking BMP signaling via noggin significantly enhances remyelination following demyelination *in vivo* (Sabo et al., 2011). While these studies firmly identify BMP signaling as refractory to remyelination, the fact that noggin promiscuously inhibits multiple BMPs, and thus signaling through several receptor classes, ultimately means that the molecular mechanisms mediating this effect remain to be elucidated. In this study, we took advantage of pharmacological developments in small-molecule inhibitors of the TGF-β signaling pathway and adopted an approach more specific to BMP4/BMPRI signaling (Cuny et al., 2008). LDN-193189 primarily inhibits BMPRIA and BMPRIB, with some inhibition of ACVRL1 (ALK1) and ACVR1 (ALK2) demonstrated in C2C12 osteoblast and chondroblast cell lines (Boergermann et al., 2010). The mechanism of inhibition involves competitive binding of the compound to the kinase domain of the type I subunits, preventing phosphorylation of downstream SMAD molecules and restricting the signaling cascade (Boergermann et al., 2010). Concordant with previous studies (Sabo et al., 2011; Karni A, Amir Levi Y, Urshansky N, Bernadet-Fainberg K (2013) World Intellectual Property Organization international patent application, PCT/IL2013/050503), here we have shown that inhibiting BMP4/BMPRI signaling with LDN-193189 significantly increased remyelination after central demyelination. This beneficial effect is achieved via selectively promoting OL differentiation, as evidenced by significantly more mature OLs after LDN-193189 administration, whereas the number of other glial cells such as astrocytes and microglia remained unchanged. This is also supported by the analysis of cultured primary OPCs in which LDN-193189 significantly potentiated OL differentiation and their subsequent myelination, and, importantly, blocked the astrogliogenic effect of BMP4 on OPCs.

It is interesting that LDN-193189 did not exert any significant effect on astrocytes during remyelination *in vivo,* whereas in our previous studies noggin infusion significantly inhibited the proliferation of GFAP^+^ astrocytes (Sabo et al., 2011; Wu et al., 2012). One consideration regarding the different astroglial effect is likely the timing of infusion. In this study, LDN-193189 was administered following a 5 week cuprizone challenge (i.e., the first week after cuprizone was withdrawn) to assess its effect on early myelin repair. However, in our previous studies, noggin was infused into the murine corpus callosum during the final third of a 6 week cuprizone challenge, when there is ongoing demyelination (Sabo et al., 2011; Wu et al., 2012). Thus, the role of BMP4 in relation to astrocytes may be proliferative in the context of acute CNS injury and be more apparent earlier in the course of disease. Potentially, astrocyte proliferation and gliosis may be modified by inhibiting BMP4 signaling activity at specific stages during demyelination and remyelination. Collectively, the results of this study, together with our previously published work, indicate that the major role of BMP4 is in promoting astrogliogenesis/astrocyte proliferation when there is active demyelination, but that it has relatively little effect on astrogliosis during remyelination following CNS injury. Additionally, the differential effects of noggin and LDN-193189 on OPCs may be due to the broader inhibitory effect of noggin. During remyelination, we found that LDN-193189 inhibition of BMP4/BMPRI/SMAD signaling selectively promotes OPC differentiation but has no effect on the generation of astrocytes. In contrast, studies using noggin to inhibit the generation of astrocytes may be achieving this through by inhibiting other BMP signaling pathways. This also is supported by *in vitro* evidence, in which noggin inhibits astroglial production *in vitro* (Grinspan et al., 2000), whereas in this study, we found that LDN-193189 or deleting BMPRIA receptor exerted little effect on astrocytes in OPC cultures where exogenous BMP4 is absent (although this may also be due to subtleties in culturing conditions). Our results together with previous data suggest that the influence of BMP4 signaling effects on OPCs is context dependent, promoting astrogliogenesis when there is active demyelination while inhibiting the differentiation of OPCs during remyelination following CNS injury.

### BMP4 signals via BMPRIA in OPCs to inhibit oligodendrocyte differentiation and myelination

Consistent with previous studies (Grinspan et al., 2000; See et al., 2004), we found that exogenous BMP4 promoted the majority of OPCs to differentiate into GFAP^+^-expressing astrocytes, while inhibiting BMP4/BMPRI signaling using LDN-193189 before BMP4 exposure is sufficient to block this effect and enhance myelination *in vitro*. Transcriptional analysis of OPCs revealed that LDN-193189 significantly downregulated the expression of *Id* family genes including *Id4*, which strongly inhibits oligodendrocyte differentiation *in vitro* (Samanta and Kessler, 2004). The resulting culture environment was such that astrogliogenesis was mostly inhibited, but residual BMP4 signaling activity prevented full differentiation of OPCs into mature OLs. It is speculated this may be due to the following two separate mechanisms: an Id4-mediated sequestering of OL transcription factor OLIG2; and synergy of BMP4-activated SMADs with the astrogliogenic pathway JaK–STAT (Janus kinase–signal transducer and activator of transcription). The action of LDN-193189 likely affects both pathways, as BMP4-induced phosphorylation of SMADs occurs upstream of both mechanisms. Different minimum thresholds of SMAD activation for each mechanism may mean that LDN-193189 has varying efficacy for inhibiting the separate effects of BMP4 signaling in OPCs. Here, we note again that *in vivo* we did not observe decreased GFAP^+^ immunostaining in mice infused with LDN-193189 following cuprizone challenge in the corpus callosum compared with vehicle-infused mice. Thus, the environmental context in which OPCs are interacting with BMP4 likely influences the specific mechanism of action directing differentiation of these cells. Notably, BMP4 treatment *in vitro* exerted a marked inhibitory effect on the expression of MBP proteins, while a relatively less robust effect was observed on MBP transcription. The precise reason behind this relatively different transcriptional and translational regulation of MBP is unclear, but suggests that BMP4 signaling exerts greater influences that target translational regulation of MBP expression. Gene function is ultimately determined by the level of protein expression. In our study, the strong effect that BMP4 exerts on suppressing MBP expression is consistent with its marked influence on inhibiting the differentiation of OPCs into mature oligodendrocytes.

Data obtained from the myelinating cocultures was in accordance with that obtained from the OPC monocultures, with BMP4 decreasing and LDN-193189 increasing myelination, respectively. Importantly, LDN-193189 blocked the inhibitory effect of BMP4 on myelination *in vitro* (See et al., 2004). One interesting observation was the significantly higher number of MBP^+^ myelinated axonal segments in cocultures treated with LDN-193189 compared with controls. This is likely due to the increased levels of endogenous BMP4 expressed by neurons and OPCs in the coculture setting. Indeed, OPCs themselves express a high level of BMP4 as they begin to differentiate (Zhang et al., 2014).

Historically, related but individual roles for BMPRIA and BMPRIB have been well identified in the regulation of various aspects of chondrogenesis and osteogenesis (Lin et al., 2016). Precisely understanding the differential influences that BMPRIA and BMPRIB receptor signaling exerts in the context of oligodendrocyte differentiation is a key step toward identifying the most suitable therapeutic targets for promoting myelination and remyelination. However, the effects of BMP4 signaling on oligodendrocyte differentiation have been inconsistent in the field, largely due to the complexity in the nature of BMP4 signaling, the genetic tools being used to target mixed cell lineages, and a diverse range of the ages and regions of the animals being analyzed. Given that global genetic knockout of BMP4 and its receptors is embryonic lethal (Mishina et al., 1995; Winnier et al., 1995), conditional genetic ablation driven by the expression of lineage markers offers a more nuanced approach to understanding BMP4 signaling in oligodendrocyte development. Previous to this study, See et al. (2007) used Cre-*loxP*-mediated transgenic excision of the *Bmpr1a* gene from cells expressing BRN4, a broad neural transcription factor activated in early embryogenesis. This was crossed with a conventional *Bmpr1b* KO mouse to generate mice with a *Bmpr1a-Bmpr1b* double KO in the neural tube by embryonic day 10 (E10.5). This leads to loss of BMPRIA/BMPRIB function in all subsequent spinal cord and hindbrain cells, causing several developmental defects and lethality at P0. Cultures of *Bmpr1a-Bmpr1b* double KO OPCs did not display phospho-SMAD1/5/8 immunoreactivity when treated with 50 ng/ml BMP4, suggesting a complete loss of the SMAD-dependent BMP4 signaling pathway in these mice. While the number of astrocytes in the spinal cord decreased at P0 compared with controls, disrupted BMP4 signaling through BMPRIA/B does not appear to affect the total number of spinal cord OPCs. Intriguingly, while the number of immature O4^+^ oligodendrocytes was unchanged, the number of mature oligodendrocytes expressing common myelin proteins, including MBP, was reduced at P0. Counterintuitively, this suggests that some level of BMP4 signaling through BMPRIA/B is required for oligodendrocyte maturation in the spinal cord and hindbrain (See et al., 2007), either through a direct effect or in combination with other synergistic pathways regulating oligodendrocyte development. Importantly, the lack of BMP4 signaling did not appear to affect the number of OPCs specified, conflicting with previous research indicating an inhibitory effect on OPC specification from neural stem cells *in vitro* (Gross et al., 1996) and in overexpression studies *in vivo* (Gomes et al., 2003).

A further study by Samanta et al. (2007), deleted BMPRIA only from neural precursor cells expressing OLIG1 from E13.5 in the neural tube, which can differentiate into neurons, astrocytes, or oligodendrocytes. This did not affect the subsequent number of OPCs at birth or at P20 (Samanta et al., 2007). However, at P20, there was an increase in mature oligodendrocytes in the BMPRIA KO group; this was at odds with the previous study, where mature oligodendrocytes were reduced at the much earlier time point. This study did not discount the possibility of increased compensatory signaling through BMPRIB, as phospho-SMADs 1, 5, and 8 were still detected. A third study by Araya et al. (2008) deleted *Bmpr1a* in *Emx-1-Cre*-expressing neural stem cells (NSCs) of the murine telencephalon. These cells develop into neurons, astrocytes, and oligodendrocytes in the telencephalon, with *Cre* recombination occurring at the peak of neurogenesis but preceding gliogenesis in the mouse. It was found that subsequent astrocytes derived from these NSCs aberrantly expressed vascular endothelial growth factor at P10, leading to the disruption of cerebrovascular angiogenesis as well as impaired blood–brain barrier formation (Araya et al., 2008). Interestingly, while previous studies using *Olig1*-*Cre*-driven *Bmpr1a* deletion showed increases in mature O4^+^ oligodendrocytes at P20, no differences in O4^+^ cells were observed at P20 in this study. In addition, compared with the earlier study deleting both *Bmpr1a* and *Bmpr1b* from BRN4-expressing cells in which GFAP^+^ astrocytes are reduced, no such decreases were observed here.

In summary, embryonic overexpression of BMP4 before or during gliogenesis clearly decreases subsequent oligodendrogliogenesis; the inhibition of BMP4 signaling embryonically using noggin has the opposite effect and increases the number of oligodendrocytes. However, See et al., demonstrated that inhibiting BMP4–SMAD signaling by deleting BMPRIA/B before OPC specification reduces the number of mature oligodendrocytes at P0 (See et al., 2007). Importantly, this was not due to reduction in the number of OPCs specified, as no changes in the number of OPCs were detected. Additionally, the study by Samanta et al. (2007) found that reduction, but not complete suppression, of BMP4 signaling through BMPRIA deletion in E13.5 neural precursor cells has no effect on the number of OPCs at P0. However, deleting BMPRIA at E13.5 increases mature oligodendrocyte number by P20. The reasons for this remain unclear. However, observations from all three studies suggest that BMP signaling through BMPRIA/BMPRIB does not play a role in the specification of OPCs from NSCs, but has a strong negative effect on subsequent OPC differentiation (See and Grinspan, 2009). Only one study specifically targeted oligodendrocyte lineage cells using an *Olig1*-*Cre* driver; however, this targets all oligodendrocytes as well as some neuronal populations. Before our study presented here, the effect of inhibiting BMPRIA in postnatal, lineage-committed OPCs had not been examined.

Using a conditional and inducible transgenic approach to specifically ablate BMPRIA expression in OPCs, we have identified that BMPRIA has a critical role in mediating the inhibitory BMP4 signal in OPC lineage progression within the postnatal CNS. We used the *Pdgfra-CreER^T2^*driver of Cre expression to specifically ablate the expression of *Bmpr1a* in postnatally derived OPCs, rather than in neural progenitor cells, or in all oligodendrocyte lineage cells, as seen with the more commonly used *Olig2-Cre* driver. As PDGFRα is downregulated in OPCs before differentiation (Ellison and de Vellis, 1994; Zhang et al., 2014), BMPRIA expression is ablated before the differentiation process occurring. This allowed us to examine the influence of BMPRIA specifically on this process, in the absence of any confounding effects of coincident deletion in mature oligodendrocytes. We found that OPCs with a BMPRIA deletion significantly attenuated the inhibitory effect of BMP4 on OPC differentiation into mature oligodendrocytes, as seen in OPC cultures treated with LDN-193189. Moreover, we found that 4OHT-treated myelinating cocultures containing BMPRIA KO OPCs showed an increased capacity to myelinate, suggesting that inhibiting BMP4/BMPRI signaling in OPCs promotes the basal level of myelination. Noticeably, the magnitude of the effect of disrupting BMPRIA expression in OPCs was lower than that seen in experiments where the signaling of BMPRI receptors is inhibited pharmacologically using LDN-193189, both at the transcriptional and protein level. For instance, the astrogliogenic effect of BMP4 treatment (as measured by the differentiation of OPCs into GFAP-expressing astrocytes) was ∼25% less in BMPRIA KO OPCs compared with control cultures, in contrast to a near-total reduction in the LDN-193189-treated OPCs. Similarly, a greater number of OPCs differentiated into either immature or mature oligodendrocytes in the LDN-193189-treated OPCs compared with the BMPRIA-null OPCs. This differential effect may be due to the latency of the turnover and replacement of functional BMPRIA receptors. The rate of BMP receptor turnover is governed by either clathrin-dependent or caveolin-dependent endocytosis, depending on whether the BMP ligand initially binds to the type I subunit or to a preformed complex of type I/type II subunits (Sieber et al., 2009). Second, it is possible that the *Pdgfra-CreER^T2^*Cre driver used did not generate a full knockout of *Bmpr1a*. We observed residual BMPRIA protein expression in 4OHT-treated DRG/OPC cocultures using Western blotting (although this may have been contributed by DRG neurons). The original study characterizing the *Pdgfra-CreER^T2^* Cre driver found ∼45–50% successful recombination of floxed DNA regions (Rivers et al., 2008). Thus, there is likely to be remaining BMPRIA expression on the OPC cell surface that may have attenuated the observed effect of inhibiting BMPRIA signaling on OPC differentiation. Further, LDN-193189 inhibits BMPRI receptors including BMPRIA and BMPRIB, whereas BMPRIB remains active in BMPRIA KO OPCs. This finding suggests that BMPRIB may also play a role in mediating the BMP4-induced inhibitory effect on oligodendrocyte differentiation and myelination in the postnatal CNS, which warrants future investigation. Both the use of LDN-193189 and OPC-targeted transgenic ablation of BMP receptor subunits may enhance the current state of knowledge regarding the role of BMP4 signaling on embryonic oligodendrocyte development, as detailed above.

In summary, our results show that inhibiting BMP4/BMPRI signaling in OPCs promotes remyelination following myelin injury *in vivo*. This beneficial effect is likely mediated by potentiating OPCs differentiation into mature myelinating oligodendrocytes. Further, we have identified that BMPRIA in OPCs plays a critical role in mediating the inhibitory effect of BMP4 on OPC differentiation and myelination. Together, our work presented here indicates that targeting BMP4/BMPRIA signaling in OPCs is a potential strategy for enhancing remyelination following a demyelinating insult.

## Synthesis

Reviewing Editor: Alfonso Araque, University of Minnesota Decisions are customarily a result of the Reviewing Editor and the peer reviewers coming together and discussing their recommendations until a consensus is reached. When revisions are invited, a fact-based synthesis statement explaining their decision and outlining what is needed to prepare a revision will be listed below. The following reviewer(s) agreed to reveal their identity: Maria Dizon, Jayshree Samanta.

The reviewers have found merit and interest on the findings reported. I concur with the reviewers' comments expressing the interest of the study. However, they also expressed some concerns about several issues that need to be addressed and clarified. I also consider pertinent the concerns expressed.

Addressing those concerns, which will definitely improve the manuscript, is necessarily required to firmly validate the results and support the conclusions.

Specific comments of the reviewers:

Reviewer 1.

The authors test the hypothesis that inhibition of BMPR1a by LDN-193189 delivered by intraventricular injection via Alzet pump or conditional inducible deletion of BMPR1a in OPCs rescues myelin after cuprizone-induced white matter injury. Cuprizone was administered enterally for 5 weeks followed by normal diet for 0 or 1 week. LDN was administered at end of Cuprizone course for 7d. Mechanisms of injury were further explored with in vitro experiments of primary cultured oligodendroglial progenitors or oligodendrocyte-dorsal root ganglion co-cultures.

The rationale for the choice of Cre should be more extensively discussed, especially since the authors assess for an effect of recombination on astrocytes.

Overall, this is a strong set of experiments supporting the hypothesis that inhibition of BMP signaling promotes remyelination after cuprizone in the CNS. The work is thorough and possible other interpretations are adequately discussed. One weakness of the manuscript is that it is not clear whether the authors believe that BMP4 promotes a progenitor to become an astrocyte or it arrests it from differentiating. Then LDN either blocks progenitors from differentiating into astrocytes or it releases the progenitor from an arrested state and allows it to differentiate. It is possible that both types of events are occurring. The writing seems to suggest different aspects of these ideas. Thus, I believe that the authors can clarify this by proposing a model that can bring together their findings with others in the field. The model could also point out inconsistencies in the field and questions that remain to be answered.

Figure 1:

Why are the newly remyelinated axons thinner in the LDN group? “LDN-infused group had significant more myelinated axons with thinner myelin sheaths (g-ratios > 0.81, Figure 1G, p=0.035b3 303) compared to the control group. Thus, these results collectively suggest that LDN infusion significantly enhances the extent of remyelination, resulting in more remyelinating axons than control group.”

Figure 2:

While it is OK to express the results as percentages indicating that, for example, x% of Olig2 + cells are also positive for PDGFRa, this only tells part of the story. For example, in Figure 2 panel A there is a large apparent difference in the total number of PDGFRa cells in the corpus callosum of the representative panel for cuprizone (5wk) and the other panels. Thus, the authors need to also show quantified numbers of cells per unit area for the corpus callosum (i.e., density o¬f cells positive for markers). Then they need to account for differences that may affect their model.

Figure 4:

Why is the MBP staining nuclear or perinuclear in some of the panels in A? Please add arrow indicating example of immature OL morphology.

Figure 5:

It is not always clear where a MBP+ myelin segment starts and ends which would affect the quantification.

Figure 6:

The title of the legend for Fig 6, “Figure 6: Inhibiting BMP4/BMPRI signaling in OPCs alters the expression of transcription factor Id4 and 825 GFAP,” does not mention the results in panels C and D which involve MBP and Myrf. Also, the degree to which MBP and Myrf are downregulated does not seem to match that seen in the stained cells of Fig 4. Could BMP be having an effect on the translation of these proteins?

Page 22:

The authors state “Taken together, the data suggest that BMP4 inhibits OPC differentiation and their subsequent capacity to myelinate axons via signaling to BMPR1 and regulating an array of downstream signalling molecules and transcription factors in OPCs.” Do the in vitro differentiation studies support “inhibition of OPC differentiation? Or do they support the hypothesis that the OPCs are reprogrammed into astrocytes. The authors might want to restate at this point that the in vivo data of figure 3 supports the author's hypothesis.

Fig 7:

Figures indicate tamoxifen as “TMX” but legend says “TAM,” since this is 4-hydroxy tamoxifen the authors need to indicate the drug as “4OHT.”

Again, it looks like MBP is nuclear or perinuclear. Could the BMP4 be inhibiting the transport of the MBP out to the edges of the cells or otherwise inhibiting translation or maturation of the protein?

Page 22: I don't believe the data in Fig 7 panel F is q-RT-PCR. I think this is plain old PCR.

Fig 8:

The authors indicate that neurofilament is “neurofil” but in panels say that it is “NFL.” This should be changed so it is consistent.

Reviewer 2.

This study is significant in elucidating the effects of BMPR1 receptor mediated signaling pathways in remyelination. The authors sought to prove that inhibiting BMP4 signaling through Type1 receptors BMPRIA and BMPRIB could promote oligodendrogenesis by inhibiting astrogliosis. They used a combination of in vivo infusion of the BMPR1 inhibitor LDN-193189 following cuprizone mediated demyelination and in vitro treatment of oligodendrocyte progenitor cells (OPCs) with LDN-193189. In addition, they genetically ablated BMPR1a receptor in vitro in OPCs to show the specific effects mediated by this receptor. Although the manuscript has several typos, the experimental steps are easy to follow, and the progression from in vivo techniques to specific testing in vitro is clear and well organized.

However, some of the conclusions made by the authors are not supported by the data. They are described below:

1. The EM data in fig.1D-G shows that the vehicle treated mice had similar numbers of myelinated axons as the LDN infused mice after 1 week of recovery from cuprizone diet. However, the G-ratios of the LDN treated mice are higher than the vehicle treated mice suggesting less myelination (greater demyelination or less remyelination) in the LDN treated group. The authors have incorrectly concluded that the LDN group shows more remyelination based primarily on the presence of thin myelin (smaller G-ratios). An analysis of numbers of unmyelinated axons correlated with the axon caliber is essential to determine the effects on remyelination.

2. The authors have concluded that LDN promotes oligodendrocyte differentiation in vivo in fig.2, which shows an increase in Olig2+CC1 double-labeled cells in the LDN recovery group with a concomitant decrease in the OPCs. Although CC1 is a marker of mature oligodendrocyte, its expression begins well before that of the myelin proteins. Hence, expression of myelin proteins is essential to show the differentiation into myelinating oligodendrocytes, which is important for remyelination effects.

3. A minor point- In fig.3A, LDN recovery panel shows the highest GFAP immunolabeling although the quantification in fig.3C shows no effects on astrocytes.

4. In fig.4, OPCs treated with LDN in vitro do not have any effect on number of oligodendrocytes compared to controls. However, LDN treatment reduces the BMP4 induced enhanced astrocyte generation. The authors have incorrectly concluded here that LDN treatment enhances oligodendrocyte differentiation.

5. Data in fig.5 shows enhanced myelin formation in vitro, in the LDN treated OPCs compared to controls. Since LDN does not increase the number of oligodendrocytes generated from OPCs (fig.4), this suggests that each oligodendrocyte is generating more myelinating processes. The authors have not discussed this in the text. An analysis of oligodendroglial processes would be useful to corroborate this data, especially since MBP and MyRF mRNA levels do not change on LDN treatment (fig.6).

6. Fig. 7 shows that genetically removing BMPR1a receptor signaling from OPCs does not alter their differentiation into oligodendrocytes and astrocytes. However, it decreases the BMP4 induced astrocyte generation. This is similar to that observed in LDN treated OPCs in fig.4, thus confirming the effects of LDN being mediated by BMPR1a inhibition. Here again, the authors have incorrectly concluded that BMPR1a inhibition potentiates oligodendrocyte differentiation.

7. Similar to the data in fig.5, BMPR1a inhibition in OPCs increases the myelinated segments in fig.8. Again, since it does not increase the number of oligodendrocytes generated from OPCs (fig.7), this suggests that each oligodendrocyte is generating more myelinating processes. An analysis of oligodendroglial processes would be useful to corroborate this data. Here the authors have shown an increase in MBP and MOG protein levels.....although it is not clear if they have examined MOG or MAG protein since MOG is mentioned in the figure and MAG is mentioned in the text.

Overall, the authors have shown that inhibition of BMPR1a can overcome the effects of BMP4 on OPCs during development (OPCs were cultured from P5-6 mice). However, the effects of BMPR1a inhibition on remyelination in adult mice are not conclusive.
